# Interplay between drought and plant viruses co-infecting melon plants

**DOI:** 10.1038/s41598-024-66344-y

**Published:** 2024-07-09

**Authors:** J. Jiménez, V. O. Sadras, N. Espaillat, A. Moreno, A. Fereres

**Affiliations:** 1 Instituto de Ciencias Agrarias, Consejo Superior de Investigaciones Científicas, ICA-CSIC, Madrid, Spain; 2 South Australian Research and Development Institute, School of Agriculture, Food and Wine, The University of Adelaide, College of Science and Engineering, Flinders University, Adelaide, Australia

**Keywords:** Allometry, Climate change, Drought, Phenology, Water stress, Leaf temperature, Fruit production, Mixed virus infection, CMV, CABYV, Cucumovirus, Polerovirus, Climate-change impacts, Plant development, Plant physiology, Plant stress responses

## Abstract

Drought affects crops directly, and indirectly by affecting the activity of insect pests and the transmitted pathogens. Here, we established an experiment with well-watered or water-stressed melon plants, later single infected with either cucumber mosaic virus (CMV: non-persistent), or cucurbit aphid-borne yellow virus (CABYV: persistent), or both CMV and CABYV, and mock-inoculated control. We tested whether i) the relation between CMV and CABYV is additive, and ii) the relationship between water stress and virus infection is antagonistic, i.e., water stress primes plants for enhanced tolerance to virus infection. Water stress increased leaf greenness and temperature, and reduced leaf water potential, shoot biomass, stem dimensions, rate of flowering, CABYV symptom severity, and marketable fruit yield. Virus infection reduced leaf water potential transiently in single infected plants and persistently until harvest in double-infected plants. Double-virus infection caused the largest and synergistic reduction of marketable fruit yield. The relationship between water regime and virus treatment was additive in 12 out of 15 traits at harvest, with interactions for leaf water content, leaf:stem ratio, and fruit set. We conclude that both virus-virus relations in double infection and virus-drought relations cannot be generalized because they vary with virus, trait, and plant ontogeny.

## Introduction

The decupling of trophic webs is a conspicuous effect of global change^1,2^. Multiple stresses are often non-additive and largely unpredictable interactions underlie ecosystem complexity^3^. Worldwide, many cropping areas are becoming drier and hotter^4^, thus facilitating the establishment of pest and pathogens into previously unsuitable regions^5,6^. Global warming is predicted to accelerate insect development, causing earlier and prolonged colonization of globally important insect pests in mid latitudes, with projected rates of increase in insect damage from 10 to 25% per °C^7^.

Hemipteran insects are expected to expand their geographical distribution and cause more damage in future climates^7,8^. Aphids damage plants directly by feeding, and indirectly as vectors of plant viruses^9^. Plant virology deals mostly with single viral infections even though mixed infections could be considered the rule more than exception^10^. Many vectors of plant viruses are polyphagous and capable of transmitting more than one virus to the same plant^11^. Inter-virus interactions are relevant because mixed infections are common in nature and agriculture with consequences for viral pathogenesis, evolution, and control^12–14^.

Plant dry matter allocation and phenological development are ecologically and evolutionary critical drivers of plant fitness and crop yield^15,16^. Tolerance to cucumber mosaic virus (CMV) of *Arabidopsis thaliana* varied among allocation types whereby long-lived genotypes with low reproductive allocation were more tolerant than short-lived genotypes with high seed-to-biomass ratio^17^. Tolerance in the former group partially associated with delayed flowering^17^. In a factorial experiment where cowpea was infected with CMV, blackeye cowpea mosaic virus (BlCMV), or both, double-virus infection reduced the leaf:stem ratio by half in comparison to controls, with no effect of single virus infection^18^.

The impact of viruses on droughted plants is important in the context of water scarcity. Infection with wheat streak mosaic virus (WSMV) reduced shoot biomass, grain yield, and water use efficiency of plants (biomass per unit water use) and crop (biomass per unit rainfall + irrigation) in hard red winter wheat (*Triticum aestivum*) cv. Karl 92, a susceptible cultivar^19^. WSMV caused a threefold reduction in wheat root biomass compared to a twofold reduction in shoot biomass of well-watered plants in the glasshouse, and impaired water uptake in the field^19^. Singly infected beets with brome mosaic virus (BMV), cucumber mosaic virus (CMV), tobacco mosaic virus (TMV) and tobacco rattle virus (TRV), improved tolerance to drought by increasing osmoportectants and antioxidant levels in the infected plants^20^. The fungus *Polymyxa betae* transmits two soilborne viruses of sugar beet, beet necrotic yellow vein virus (BNYVV) and beet soilborne mosaic virus (BSBMV); a factorial experiment combining non-inoculated control, plants infected with BNYVV, BSBMV or both, and three water regimes showed all virus treatments reduced water uptake in well-watered plants, but not in plants under drier soil^12^. In a field experiment, disease symptoms increased with increasing frequency of irrigation, and all virus treatments reduced crop water use efficiency (beet root biomass per unit evapotranspiration)^12^. In a study with grapevines grown from cuttings planted in pots outdoors, whole-plant water use efficiency responded to the interaction between water regime and grapevine leafroll-associated virus 3 (GLRaV-3) in only one out of four cases, whereby water use efficiency increased in virus-infected plants under water stress, with no variation in well-watered plants^21^.

Both inter-virus relationships in mixed infections and virus-drought relations have attracted substantial research effort, and a quantitative review captured antagonistic, additive, and synergistic inter-virus relationships in double infections whereas virus-drought relationships are dominantly additive or antagonistic, reinforcing the notion that viruses have neutral or positive effects on droughted plants, or that drought enhances plant tolerance to viruses^22^. However, this interpretation partially stems from experiments where virus-infected plants are subjected to water stress after virus infection; the sequence of treatments, namely virus infection followed by drought or drought followed by virus infection influences the outcome of the relationships. The interplay between inter-virus relationships in mixed infections and drought, the focus of this study, remains largely unexplored^22^.

With melon (*Cucumis melo*) as a model plant that is commonly infected by multiple viruses^23^, we established a full factorial experiment that sequentially combined two water regimes (well-watered, water-stressed) followed by four aphid-inoculated virus treatments: 1) mock-inoculated controls infested with non-viruliferous aphids, 2) infection with the persistently transmitted cucurbit aphid-borne yellow virus (CABYV, genus *Polerovirus*), 3) infection with the non-persistently transmitted cucumber mosaic virus (CMV, genus *Cucumovirus*) and 4) infection with both CMV and CABYV. Virus treatments were established with inoculation by *Aphis gossypii* Glover, an efficient vector of both CMV and CABYV^24^. Plants were grown until fruit reached size and sugar concentration conforming market standards. Our aim was to test two hypotheses with a focus on multiple plant traits (leaf and whole-plant scale, vegetative and reproductive): (1) the relation between CMV and CABYV is additive and, (2) the relationship between water stress and virus infection is antagonistic, i.e. water stress primes plants for enhanced tolerance to virus infection.

## Results

### Virus infection and visual symptoms

**Figure 1:**
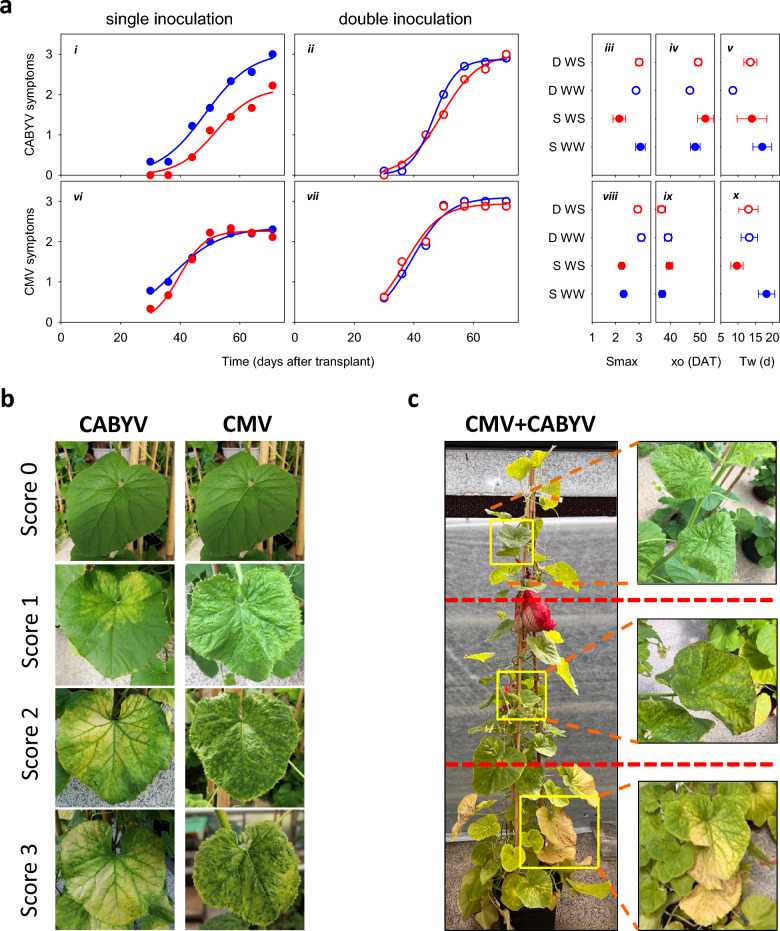
(**a**) Dynamics of (i, ii) CABYV and (vi, vii) CMV foliar symptoms in plants with (i, vi) single and (ii, vii) double infection in well-watered (blue) and water-stressed treatments (red). Curves are sigmoid with three parameters: (iii, viii) Smax, representing the maximum symptom score; xo, (iv, ix) the inflection point; (v, x) the transition window Tw, a derived parameter representing the time between 0.25 and 0.75 Smax; a large transition window associates with a low rate of symptom development. In iii-v, viii-x, S: single infection; D: double infection; WW: well-watered; WS: water stressed; error bars are two standard errors, and are not shown when smaller than symbol. (**b**) Scale 0–3 used to score virus symptoms. (**c**) Double CMV-CABYV–infected plant showing symptom distribution for CMV in younger, upper leaves and CABYV dominantly in older, bottom leaves.

Four plants were excluded from the analysis due to failure of virus infection and three plants were excluded due to mechanical contamination with CMV. One plant initially assigned to well-watered mock-inoculated control, one initially assigned to water-stressed mock-inoculated control, and one initially assigned to well-watered CABYV were unintentionally infected with CMV. According to ELISA, one plant in water-stressed CMV, one in water-stressed CABYV and two plants in water-stressed CMV + CABYV escaped infection Therefore, the number of replicates per treatment was 10 for each: well-watered CMV + CABYV and well-watered CMV; 9 for each: well-watered control, water-stressed control, water-stressed CMV, well-watered CABYV, and water-stressed CABYV; and 8 for water-stressed CMV + CABYV. Figure 1b,c shows typical symptoms in plants with single and double virus infection and the visual scale for scoring symptoms. Based on the 73 plants with reliable treatments (n ≥ 8 per treatment), we fitted three-parameter sigmoid curves to capture the trajectories of disease symptoms according to the symptom scale previously described (Fig. 1a; i, ii, vi, vii; Adj R^2^ ≥ 0.95, *p* < 0.0001; Supplementary Table S1).

In CABYV-infected plants, water deficit slowed down symptom development (Fig. 1a; i); the inflection point of the curve was delayed 4 d, and the maximum symptom score was 2.2 compared to 3.1 in well-watered plants (Fig. 1a; iii, iv). Scale used for virus symptomatology scoring and also a more visual comprehension of both virus symptoms dynamics in CMV + CABYV-infected plants is indicated in Fig. 1b,c. Also, a picture of the well-watered plants within each virus treatment at harvest is shown in Fig. 7c. Symptom severity was delayed and was less intense for water stressed-CABYV infected plants throughout the experiment in comparison to the well-watered counterparts (Fig. 1a; i). However, the effect of water regime was not apparent for the symptoms of CABYV in double-infected plants (Fig. 1a; ii). The progression of CABYV symptoms in well-watered plants was faster in double infected plants than in plants with single infection as shown in a two-fold difference in the transition width, from 17.0 d under single inoculation to 8.5 d in co-infected plants (Fig. 1a; v).

In CMV-infected plants, water deficit delayed the onset of symptom development (Fig. 1a; vi). Water deficit caused a two-fold increase in the rate of symptom development, i.e., the transition width of water-stressed plants was 9.7 d compared to 18.3 d in well-watered ones (Fig. 1a; x). Faster rate fully cancelled the later onset, thus a similar maximum score for CMV-infected plants irrespective of water regime (Fig. 1a; viii, ix). Double-infected plants increased maximum CMV symptom score to 3.0 in comparison to 2.3 in single-infected plants (Fig. 1a; viii).

### Leaf water potential

**Figure 2:**
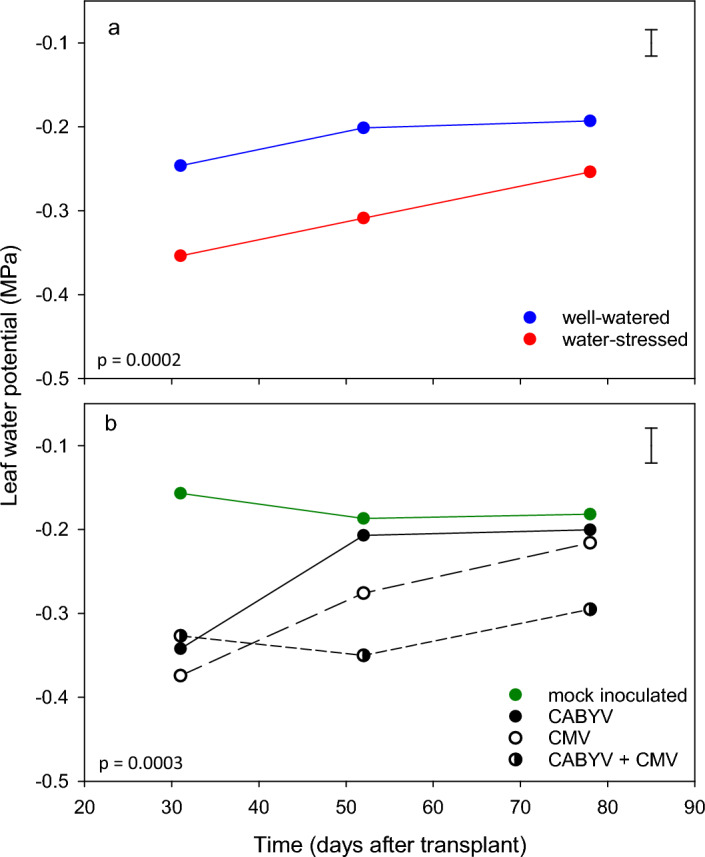
Midday leaf water potential in melon plants in response to (**a**) water regime and (**b**) virus treatment. Leaf water potential varied with water regime (*p* = 0.0002) and virus treatment (*p* = 0.0003) with no effect of interaction (*p* = 0.25). Symbols are least squares averages and error bars are two standard errors.

Midday leaf water potential varied with time, water regime (Fig. 2a), and virus treatment (Fig. 2b), but not with the two- and three-way interactions (Supplementary Table S2). Leaf water potential was 0.06–0.10 MPa lower in water-stressed plants than in well-watered controls (Fig. 2a). At 31 days after transplant (DAT), leaf water potential in virus-infected plants was lower than in controls (Fig. 2b). From 31 to 78 DAT, the trajectories of leaf water potential of CABYV and CMV-infected treatments converged with that of the mock-inoculated control, whereas leaf water potential remained lower in double-infected plants (Fig. 2b).

### Leaf temperature

Bottom-leaf temperature varied with time, water regime, virus treatment, and with all the two-and three-way interactions except for that between time and water regime (Supplementary Table S2). In mock-inoculated, well-watered controls, leaf temperature increased over time from 19.4 ± 0.56 °C at 9 DAT to 23.2 ± 0.79 °C at 73 DAT. Across sources of variation, leaves of water-stressed plants were 0.48 °C hotter than those in their well-watered counterparts.

**Figure 3:**
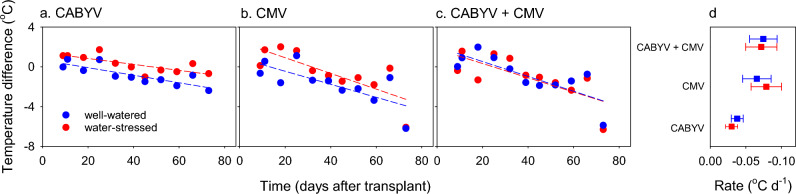
Time-course of bottom-leaf temperature in well-watered and water-stressed plants infected with (**a**) CABYV, (**b**) CMV and (**c**) CABYV + CMV. Temperature is the difference with well-watered mock-inoculated control. Lines are least square regressions (0.70 ≥ adj R^2^ ≥ 0.55; *p* ≤ 0.011); quadratic models did not improve the fit. (**d**) Rate of change in leaf temperature calculated as the slope (± s.e.) of the linear regressions in (**a**–**c**).

Figure 3 captures the intricate interactions between virus, water regime and time. Nine days after virus inoculation, all virus treatments increased leaf temperature in comparison to the well-watered, mock-inoculated controls (Fig. 3abc). With time, leaves of all virus-infected plants were gradually cooler. The slopes of linear regressions highlight that CMV in both single and double infection had a larger cooling effect than CABYV (Fig. 3d).

The intercepts of the regressions partially capture the interaction between virus and water regime: in comparison to well-watered plants, leaves of water-stressed plants were 0.8 °C hotter under CABYV infection, and 1.6 °C hotter under CMV. The trajectories of leaf temperature overlapped for well-watered and water-stressed plants in double infected plants, indicating that double infection overrode the effect of water regime on leaf temperature (Fig. 3c). Top-leaf temperature varied with time, water regime, virus, and two-way interactions (Supplementary Table S2); five dates of measurement, in comparison to 11 measurements for the bottom leaf, precluded further model fitting for top-leaf temperature.

### Leaf greenness

**Figure 4:**
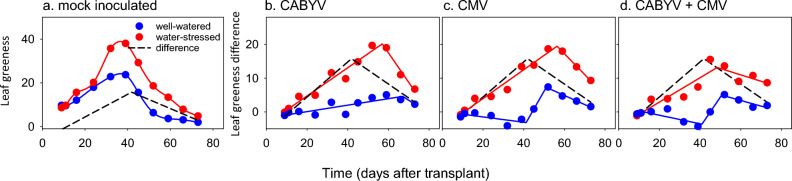
(**a**) Time course of bottom-leaf greenness (unitless) in well-watered (blue) and water-stressed (red) mock-inoculated controls. Leaf greenness varied with time, virus and time x virus interaction (all *p* < 0.0001), water regime (*p* = 0.0005), water x virus interaction (*p* = 0.0002), and the three-way interaction time, virus, water (*p* = 0.0018), with no effect of time x water interaction (*p* = 0.71). Points are least squares averages and curves are splines. The dashed black line is a piecewise, 2 segment model fitted to the difference between water-stressed and well-watered (adj R^2^ = 0.88, *p* < 0.0001). Difference in leaf greenness between plants infected with (**b**) CABYV, (**c**) CMV and (**d**) CABYV + CMV and well-watered, mock-inoculated control. In (b-d) the red and blue lines are piecewise, two (0.56 ≥ adj R^2^ ≥ 0.95; *p* ≤ 0.01) or three (0.72 ≥ adj R^2^ ≥ 0.82; *p* ≤ 0.04) segment models, and the dashed black line from (**a**) is drawn for comparison.

Bottom-leaf greenness varied with time, water regime, virus treatment and the interactions between time and water regime, and time and virus treatment (Supplementary Table S2). In mock-inoculated controls, leaf greenness increased to a peak at ~ 39 DAT; water-stressed leaves were greener than those in well-watered plants (Fig. 4a).

In CABYV-infected plants, the increase in greenness with water stress (red line in Fig. 4b) copied the effect of water stress in the absence of virus (black line in Fig. 4b), but this effect persisted for an additional two weeks, i.e., the difference peaked at 42 DAT in the absence of virus and at 57 DAT with CABYV. In well-watered plants, CABYV increased leaf greenness slightly in comparison to mock-inoculated controls (blue line in Fig. 4b). In single CMV-infected plants, the increase in greenness with water stress (red line in Fig. 4c) copied the effect of water stress in the absence of virus (black line in Fig. 4c), but this effect persisted for an additional 10 d. In double infected plants, the increase in greenness with water stress (red line in Fig. 4d) copied the effect of water stress in the absence of virus (black line in Fig. 4d) with no apparent shift in persistence of this effect. In well-watered plants, CMV on its own or combined with CABYV affected greenness in three-phases in relation to mock-inoculated controls. First, leaf greenness declined below the level of mock-inoculated treatments until ~ 40 DAT (first segment, blue line Fig. 4cd). Secondly, leaf greenness increased until ~ 51 DAT (second segment, blue line Fig. 4cd), and, third, it declined afterwards (third segment, blue line in Fig. 4cd). Top leaves were greener under water stress than in well-watered plants and greenness varied with time but not with virus treatments or interactions (Supplementary Table S2).

### Leaf area

**Figure 5:**
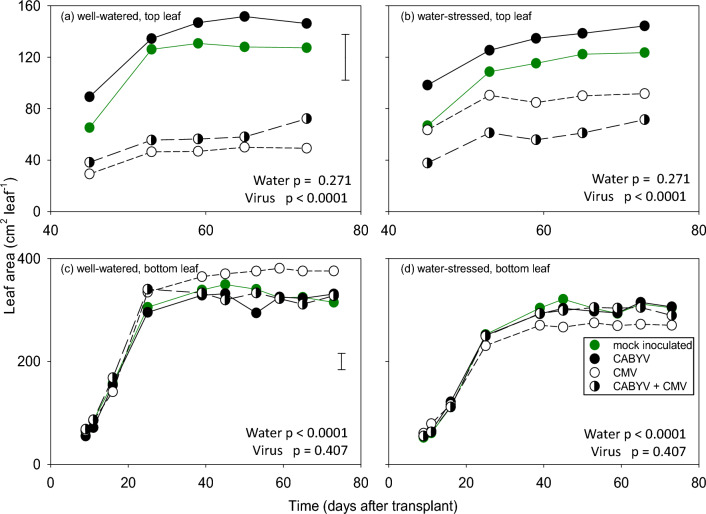
Dynamics of leaf expansion in top (**a**, **b**) and bottom (**c**, **d**) leaves of (**a**, **c**) well-watered and water-stressed (**c**, **d**) melon plants inoculated with CABYV, CMV, CABYV and CMV, and mock-inoculated controls. Top leaf area varied with time, virus (both *p* < 0.0001) and the interaction between virus and water regime (*p* = 0.012). Bottom leaf area varied with time, water regime and the interaction between virus and water regime (all *p* < 0.0001). Values are least squares means and error bars are two standard errors. Note the difference in both *x* and *y* scales between top and bottom leaves.

Top-leaf area varied with virus and with the interaction between virus and water regime (Supplementary Table S2); in well-watered plants CMV and double infection reduced leaf area similarly whereas in water-stressed plants, the reduction was larger in double-inoculated plants (Fig. 5a,b). Bottom-leaf area varied with water regime and with the interaction between virus treatment and water regime (Supplementary Table S2). Leaf area did not vary between mock inoculated controls, and plants infected with CABYV or both CABYV and CMV; single infection with CMV slightly increased leaf area in well-watered plants and had no effect under water stress (Fig. 5c,d).

### Dynamics of flowering and fruit set

Number of female flowers varied with time, water regime, virus treatment and all three two-way interactions (Supplementary Table S3).

**Figure 6:**
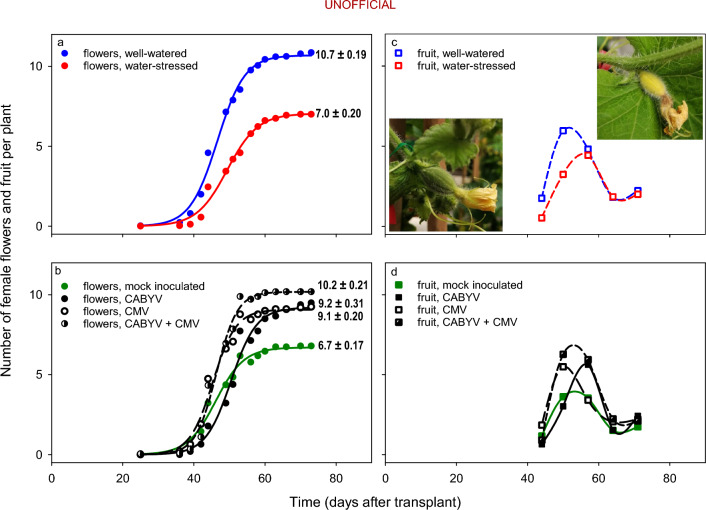
Dynamics of flowering and fruit set in melon plants in response to (**a**, **c**) water regime and (**b**, **d**) virus treatment. Data points are least squares averages. In (**a**, **b**) curves of cumulative flower number are 3-parameter sigmoidal with 0.99 ≤ adj. R^2^ ≤ 0.97, *p* < 0.0001; numbers next to curves are the maximum number of flowers (± s.e.) from the fitted model. In (**c**, **d**) curves are spline. In (**c**) photos are similar-age fruit shortly after pollination highlighting (left) viable fruit and (right) yellowing fruit in the process of aborting.

Sigmoidal models captured these complex effects on the dynamics of flowering (Fig. 6a, b). Water stress reduced the rate of flowering leading to a 35% reduction in final flower number compared to well-watered plants (Fig. 6a). In comparison to mock-inoculated controls, CABYV infection delayed the onset and increased the rate of flowering, leading to a 36% increase in final flower number (Fig. 6b). Infection with CMV did not change the onset and increased the rate of flowering to a lesser extent than CABYV, leading to a similar final number of flowers in both virus treatments. Plants with double virus infection had the highest rate of flowering leading to a 52% increase in final flower number compared to their mock-inoculated counterparts.

The dynamics of fruit set had two phases. First, fruit number increased up to a peak and declined in a second phase dominated by fruit abortion (Fig. 6c, d). The rate of increase in fruit number in the first phase paralleled the effects of both water regime and virus treatment; for example, well-watered plants featured higher rate of fruit accumulation and a higher and earlier peak, with a longer abortion phase leading to similar final fruit number (Fig. 6c). Similarly, the interplay between rate and duration of the first phase led to similar final fruit number in all four virus treatments at 73 DAT (Fig. 6d).

### Traits at harvest

**Figure 7:**
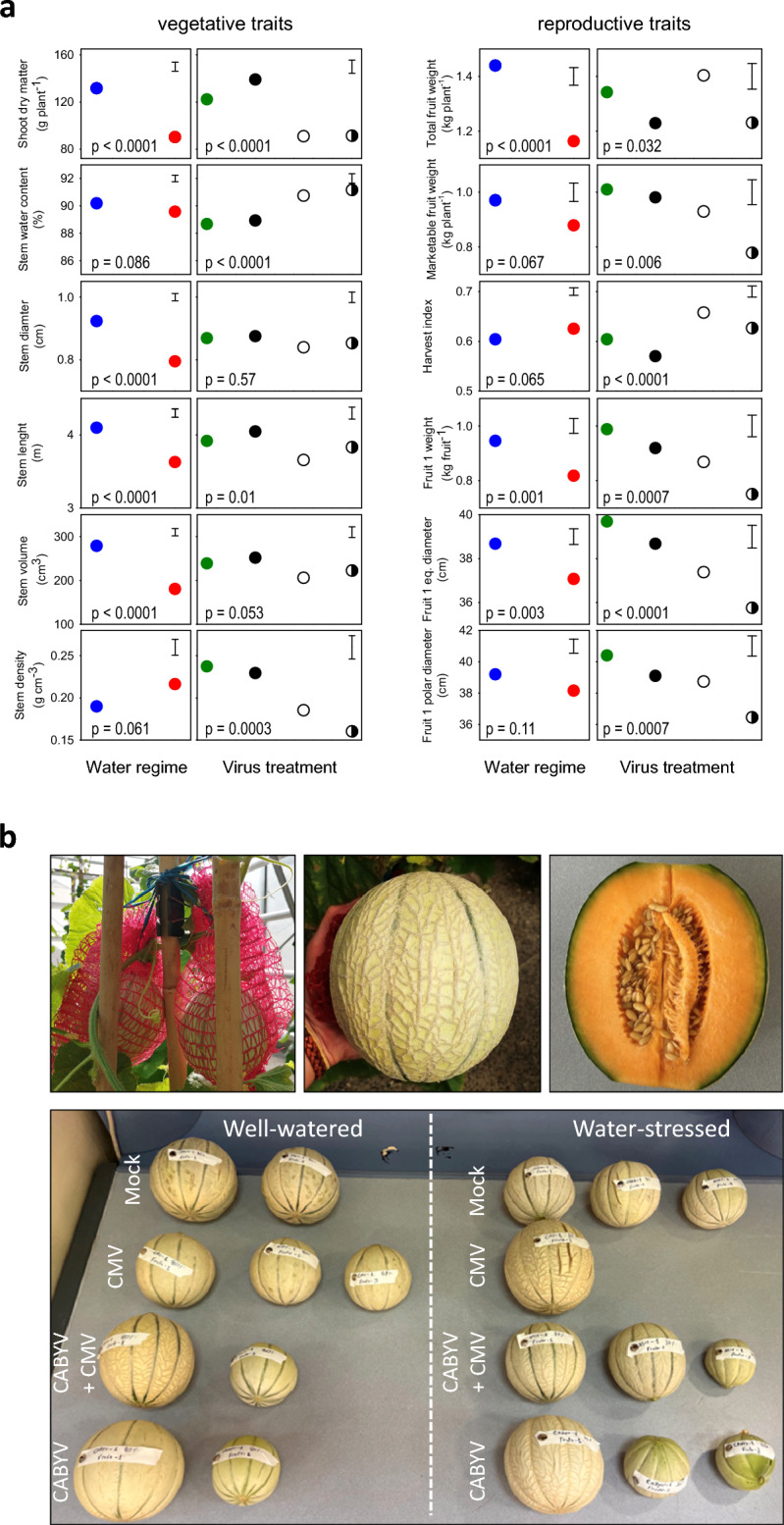

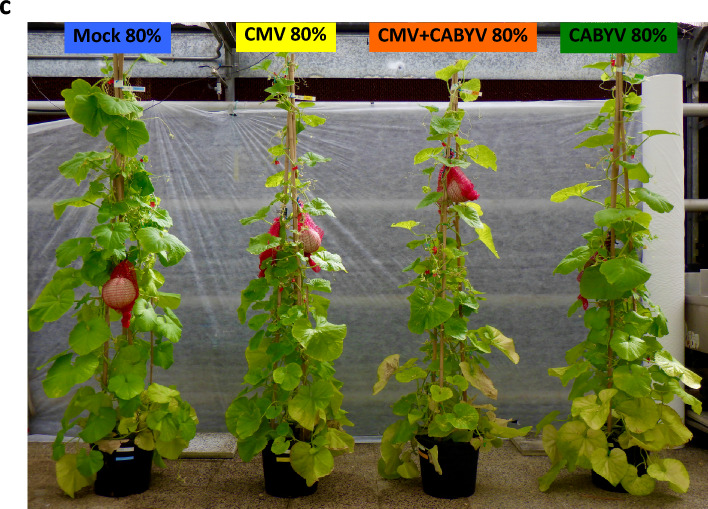
(**a**) Vegetative and reproductive traits at harvest that varied with water regime and virus treatment, but not with the interaction between water regime and virus treatment. Water regimes are well-watered (blue) and water-stressed (red). Virus treatments are mock inoculated (green), CABYV (black), CMV (white) and double inoculated (half black, half white). Values are least squares means and error bars are two standard errors (top right corner) for comparison of water regimes or virus treatments. (**b**) Top-left: fruits were supported with mesh-bags attached to the sticks; top-middle: melon harvested at ripping stage; top-right: mid-section of a ripe melon that reached marketable standards for size and sugar concentration. Bottom: fruits of the first plant for each treatment (1st replicate). On the left, fruits from the well-watered plants (from top to bottom: mock, CMV, CMV + CABYV, CABYV); on the right, fruits from water-stressed plants (from top to bottom: mock, CMV, CMV + CABYV, CABYV). (**c**) Illustration of plant size and virus symptoms at harvest in well-watered plants (80% pot capacity).

**Figure 8:**
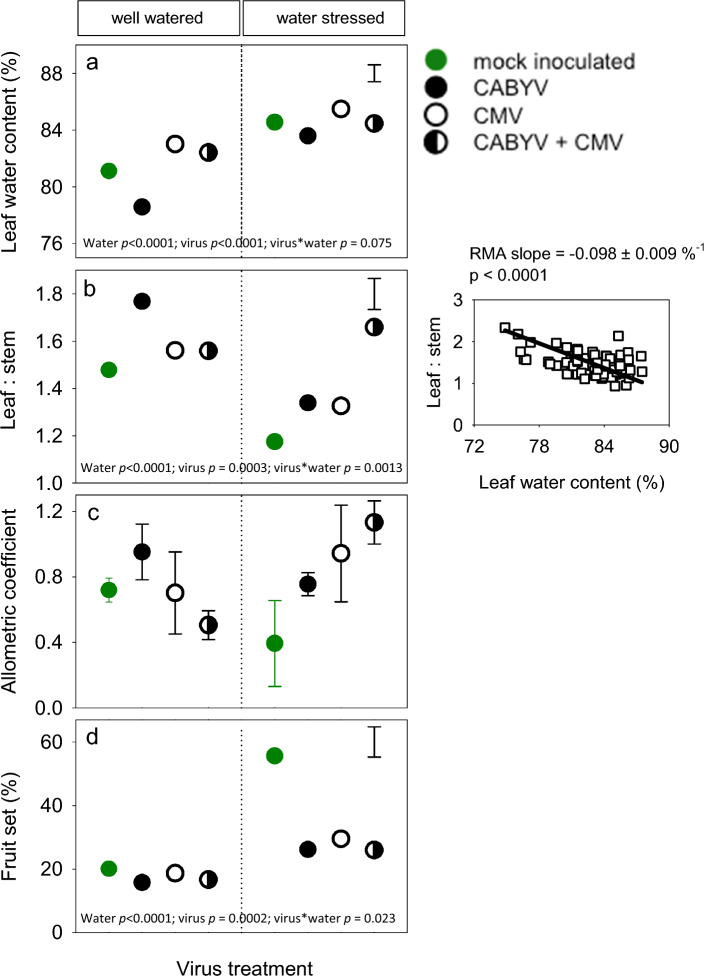
Traits at harvest that varied with water regime, virus treatment, and the interaction between water regime and virus treatment. (**a**) Leaf water content. Two traits to quantify leaf:stem partitioning (**b**) ratio, and (**c**) allometric coefficient. (**d**) Fruit set, i.e. percentage of flowers setting fruit. In (**a**, **b**, **d**) points are least squares means and error bars are two standard errors. In (**c**), allometric coefficients are the slope (± s.e.) of the reduced maximum axis (RMA) regression between leaf and stem dry matter in a log–log scale. Inset in (**b**) is the relationship between leaf:stem ratio and leaf water content, where each point is a single plant; the line is the RMA regression accounting for error in both x and y. *p* from the fitted models are shown at the bottom of the graphs for the effect of water, virus, and their interaction (virus*water).

The interaction between water regime and virus treatment affected 3 out of 15 traits (Supplementary Table S4). Hence, depending on the interaction, this section focuses on the individual effects of water and virus treatments (Fig. 7) or their combined effect (Fig. 8).

#### Vegetative growth, partitioning and morphology

The effect of water regime and virus inoculation was additive, i.e., *p* ≥ 0.25 for the interaction, for shoot biomass, stem water content, and the diameter, length, volume, and density of stems (Supplementary Table S4). Water stress increased stem density and reduced shoot biomass, stem water content, and the diameter, length, and volume of stems in comparison to well-watered plants (Fig. 7a, left panels; (Supplementary Table S4). Infection with CMV, individually or in combination with CABYV, reduced shoot biomass and stem dimensions, and increased stem water content (Fig. 7a, right panels; Supplementary Table S4).

Two traits, leaf water content and leaf:stem ratio, varied with all three sources of variation (Fig. 8; Supplementary Table S4). Leaf water content was higher under water-stress, and the drop in well-watered plants was largest for CABYV-infected plants and smallest for double-infected plants (Fig. 8a). Water stress reduced the leaf:stem ratio, and this reduction was largest for CABYV-infected plants, and negligible for double- infected plants, with intermediate responses for mock-inoculated and CMV infected plants (Fig. 8b). Leaf:stem ratio declined with increasing leaf water content (Fig. 8d).

Allocation ratios are size-dependent, hence the need for careful interpretation^63^. To account for size dependence, we calculated allometric coefficients as the slope of the RMA regression between leaf dry matter and stem dry matter in a log–log scale. Under water stress, the allometric ratios aligned with leaf:stem ratios (Fig. 8b vs. c), i.e., leafiness was lowest in mock-inoculated plants, highest in double-infected plants, and intermediate in plants with single virus inoculation. In contrast to ratios, that *declined* with water deficit in three out of four virus treatments, the allometric coefficient was more stable and *increased* with water deficit in double-infected plants (Fig. 8d).

#### Yield and fruit traits

Marketable melons were harvested at the end of the experiment (Fig. 7b). Six out of seven yield traits did not vary with the interaction between water regime and virus treatment (Supplementary Table S4; Fig. 7a, right column). The exception was fruit set that varied with all three sources of variation (Fig. 8d). Fruit set varied with virus treatments 17–20% in well-watered plants, and increased under water stress, 2.8-fold in mock-inoculated plants compared to 1.6-fold in their virus-infected counterparts (Fig. 8d).

At harvest, plants inoculated with CMV singly or in combination with CABYV, had a slightly higher fruit number than mock-inoculated and CABYV-infected plants (2.0 vs. 1.6, s.e. = 0.14, *p* = 0.04), whereas fruit number did not vary with water regime (*p* = 0.58) and interaction (*p* = 0.64) (Supplementary Table S4). Total fruit weight and weight of marketable fruit varied additively with water regime and virus treatment (*p* < 0.0001 for water; *p* = 0.03 for virus; Supplementary Table (S4). Water stress reduced total fruit weight by 20% and marketable fruit weight by 11% in relation to well-watered plants. Infection with CABYV or CABYV and CMV caused a 10% reduction in fruit weight. Reduction in marketable yield with double virus infection, 23.5%, was larger than for single inoculations (Fig. 7a). Harvest index increased slightly from 0.60 in well-watered plants to 0.63 under water deficit. Harvest index increased in plants infected with CMV singly and in combination with CABYV.

Fresh weight, polar and equatorial diameter, and sugar content were measured in the dominant, largest fruit per plant (Supplementary Table S4; Fig. 7a). Water stress slightly reduced fruit equatorial diameter and had no effect on polar diameter. Double virus infection caused a 10% reduction in both equatorial and polar diameter. Treatments had no effect on fruit sugar content (Supplementary Table S4).

## Discussion

### Experimental setting: implications for the interpretation of results

Field crops are often water stressed, multiple virus infection is common, and the combination of drought and virus infection is therefore important. Here, we studied the interplay between CMV and CABYV in single and double infection, and the interplay between virus infection and water regime using melon as a model plant. In a single glasshouse experiment, we grew plants until marketable fruit production in contrast to many other studies with seedlings or vegetative plants^25–29^. In common with^30–32^ we established water stress before virus inoculation as opposed to studies of virus-drought relations where plants were subjected to water stress weeks after virus inoculation^20,28, 29^. Our experimental sequence of treatments corresponds to a scenario where virus infection follows the development of drought stress in association with, for example, (i) dry spells early in the season and (ii) gradual development of viral diseases^22^. Plant virus spread is a gradual process and may take several infection cycles that last many days for non-persistently transmitted viruses, including CMV in our study^33^. Moreover, this process could take even longer for persistently transmitted viruses such as CABYV, since longer time is required for virus acquisition, plus the latent period and further spread of the insect vector to neighboring plants^34^.

Growing potted plants in a glasshouse introduces artifacts that prevent extrapolation to field conditions including unrealistic root systems^35^ and unrealistic boundary layer that alters that degree of coupling between plant and surrounding air^36,37^. Nonetheless, we were able to grow plants that reached fruit size and sugar concentration with marketable standards and many seeds (Fig. 7b). Under these experimental conditions we tested the hypotheses: (H1) the relation between CMV and CABYV is additive, and (H2) the relationship between drought and virus infection is antagonistic, i.e., water stress primes plants for enhanced tolerance to virus infection.

#### Hypothesis 1

The relation between CMV and CABYV is additive.

The dynamics of leaf water potential, leaf greenness and temperature highlight that virus-virus relations can be additive, synergistic or antagonistic depending on both trait and plant ontogeny. For example, at 31 DAT, single-virus inoculation reduced leaf water potential in relation to mock-inoculated control by 0.19 MPa for CABYV and 0.22 MPa for CMV (Fig. 2); the expected reduction from an additive effect (0.19 + 0.22 = 0.41) compared with an actual reduction of 0.17 MPa, returning an actual-to-additive ratio of 0.42 (0.17/0.41), i.e. the reduction in leaf water potential was 42% of that expected from additivity in correspondence with an antagonistic relationship^22^. At 78 DAT, the actual-to-additive ratio was 2.1, highlighting the synergy between CMV and CABYV (Fig. 1). The variation of virus-virus relations affecting leaf water potential could be partially related to artifacts of experimental conditions or legitimate differences between plant-virus-drought systems^22^. In melon, a small sample of three cultivars showed a range from an anisohydric-like phenotype, with a weak coupling of stomatal closure with leaf water potential to a more typical isohydric phenotype with stomata closure contributing to the maintenance of leaf water potential^38^; virus-virus relations could therefore vary with genotype to the level of cultivar reaction to water deficit.

Comparison of traits at harvest highlight the trait-dependence of inter-virus relationship, e.g., it was synergistic for shoot dry matter (actual-to-additive ratio = 2.1), close-to-additive for water content of stem (actual-to-additive ratio = 1.06), and antagonistic for stem length (actual-to-additive ratio = 0.65). The trait-dependence of virus-virus relationship is apparent in other combinations of viruses, plant species, and growing conditions: the relationship between BSBM and BNYVY in field-grown beet was antagonistic for yield and synergistic for viral symptoms12; the relationship between BICMV and CMV was synergistic for yield and additive for stem and root growth in glasshouse-grown cowpea^18^; the relationship between CMV and WMV was antagonistic for fruit number and fruit yield, and additive for fruit flesh thickness, polar and equatorial diameter in field-grown melon^39^.

#### Hypothesis 2

The relationship between drought and virus infection is antagonistic.

The interplay between virus and drought has been extensively studied, with several works showing that viruses have neutral or positive effects on plant undergoing drought, or less frequently, that drought enhances tolerance to plant viruses^20,40, 41^. Under our experimental conditions, virus-drought relation was additive for 12 out of 15 traits at harvest, supporting the trait-dependence of the relationship; interactions were apparent for allocation traits (Fig. 7, 8). Where interactions between virus infection and drought were detected, they were mostly antagonistic, e.g., for leaf area, leaf temperature, leaf greenness, fruit setting, leaf:stem ratio and leaf water content, suggesting that drought partially attenuated some of the negative effects caused by virus infection. Therefore, we conclude that the priming effect of drought enhancing tolerance to virus infection was trait-dependent, and rare.

Water stress delayed and reduced the severity of CABYV symptoms in single-infected plants (Fig. 1). In comparison, water stress delayed the onset but increased the rate of development of CMV symptoms under single infection. Irrespective of water regime, the presence of CABYV enhanced CMV symptoms in double inoculated plants. The presence of CMV enhanced CABYV symptoms in water-stressed plants but not in their well-watered counterparts. The dynamics of symptom development was therefore virus-dependent, and varied with co-infection and water regime. One example of the mismatch between symptoms and relevant traits is the relationship between beet soilborne mosaic virus (BSBMV) and beet necrotic yellow vein virus (BNYVV), which was synergistic for disease symptoms and antagonistic for yield under the experimental conditions of Piccinni and Rush12. The relationship between pepper huasteco virus (PHV) and pepper golden mosaic virus (PepGMV), quantified with plant symptoms, was antagonistic in pepper and synergistic in tobacco and *Nicotiana benthamiana*^42^. For CMV, symptoms depend on the viral isolate, host plants, co-infection with other viruses and weather conditions^43^. In melon var. ‘Monique’, CMV symptoms were more severe in the apical stems where the top leaf measurements were conducted. Thus, lack of influence of CMV symptoms on the basal part of the plant led to no effect of the virus on the leaf area of the bottom leaf. Here, we show the virus-dependence of the virus-water interplay for foliar symptoms.

The dynamics of leaf temperature illustrate the variation in virus-drought relations with plant ontogeny. Both CABYV and CMV, in single or double infection, increased leaf temperature shortly after establishment of treatments (Fig. 3; leaf temp). We did not find reports of leaf temperature in melon plants in response to virus infection in combination with water stress. Increased temperature has been reported for infection with Barley yellow dwarf virus (BYDV; Luteovirus) in barley under field and controlled conditions, as BYDV increased temperature in infected plants in comparison to the control^44^. This is consistent with higher stomatal conductance and lower leaf water potential. With the progression of the experiment, virus-infected leaves became hotter than mock inoculated treatments. Whereas leaves of water-stressed plants were consistently hotter than those of their well-water counterparts, double-infection removed these effects. The intriguing proposition that virus infection could shift the plant phenotype from isohydric to anysohydric deserves investigation.

### Virus infection altered reproduction with biological and agronomic implications

Single infection with CMV or CABYV promoted the production of female flowers in comparison to the mock-inoculated plants, and this enhancing effect was larger in doubly inoculated plants (Fig. 6). However, the increased flower production did not increase fruit yield as rate of fruit abortion increased in infected plants (Fig. 6). All angiosperms over-produce flowers and ovules, and several explanations have been advanced to explain the evolution and persistence of this trait ^45,46^. This overproduction of flowers may compensate for the losses of developing embryos^47^, to anticipate favorable pollination and/or resources for seed production^48^, for the selective abortion of low-quality embryos^49^ and/or for uniform seed production via the selection of fertilized ovules with similar resource absorption rates^46^. In fact, we found an almost twofold increase of fruit set for water-stressed mock-inoculated plants (57.3%) in comparison to the water-stressed, virus infected plants (CMV: 30.7%; CMV + CABYV: 24.3%; CABYV 25.1%), and almost threefold for in comparison to well-watered plants across virus treatments (Mock: 19.7%; CMV: 18.8%; CMV + CABYV: 17.1%; CABYV: 15.1%) (Fig. 8d). The causes and consequences of enhanced flower production in virus-infected plants need further investigation.

Under our experimental conditions, single infection reduced the marketable fruit weight by 8.3% for CMV, 4.4% for CABYV, and 23.5% in plants infected with both CMV and CABYV (Fig. 7). The actual-to-additive ratio of 1.9 highlights the synergistic effect of mixed infection, reducing yield almost twice as much as expected from additivity. Infection with CMV reduced yield of field-grown bell-pepper by 80% when plants were inoculated 1 week after transplant, and by 30% when the virus was inoculated 7 weeks after transplant^50^. This age-dependent response is known as mature plant resistance^50,51^. When peppers were double infected with CMV and PVY the relationships between viruses shifted from antagonistic to additive with delayed inoculation indicating that mature plant resistance did not hold in double infected plants^22^. On melon plants var. ‘Piel de sapo’, the effect of double infection with CMV and WMV was antagonistic for yield under the experimental conditions of Alonso–Prados et al.^39^. These are clear examples of the host-dependence to the level of species or cultivars in response to inter-virus relationships^42,52, 53^.

## Conclusion

A literature review showed antagonistic (18%), additive (48%) and synergistic (33%) virus-virus relations in double infected plants, and antagonistic (57%) and additive (43%) but no synergistic virus-drought relations^22^. Here we found variation in both virus-virus and virus-drought relations depending on virus, trait, and plant ontogeny. Our research highlights the reduction in marketable yield with water-stress and virus infection, particularly in plants with double virus infection that reduced yield synergistically. Work is needed to establish economic injury levels to inform pest control accounting for insect vectors of plant diseases that can spread disease at very low densities.

## Methods

### Experimental setting and design

Plants were grown in a glasshouse at the ICA-CSIC in Madrid, Spain (40.43966, − 3.68727: 698 m above sea level) from May 2023 to August 2023. The experiment was a factorial with two water regimes and four virus treatments, returning eight virus-water treatments. Virus treatments were a mock-inoculated control (plants infested with non-viruliferous aphids), and plants singly infected with CMV or CABYV, and double infected-plants infected with both viruses (CMV + CABYV). Water regimes were well-watered control maintained at 80% pot capacity and water stress maintained at 30% pot capacity.

We aimed at 10 replicates per treatment but in some treatments, we achieved 8–9 replicates due to failure in virus infection, as not all inoculated plants became infected (see below). Plants were allocated to a column—row design to achieve two goals: minimizing mutual shading between columns, which were SE-NW oriented, and spreading treatments spatially for each to explore all glasshouse conditions (Supplementary Fig. S1). This row-column design is statistically and logistically superior to regular re-arrangement of pots in the glasshouse^54^.

### Aphid colonies and virus species

For virus inoculation, we used an *Aphis gossypii* colony obtained from a single virginoparous female from a population collected in Almeria (Spain) in 1998. Non-viruliferous *A*. *gossypii* colonies were maintained in melon plants cv. ‘Bazán’ (Semillas Fitó, Barcelona, Spain) and renewed fortnightly to assure newly emerged adults to run the experiments. Colonies were kept in a growth chamber with a 16 h photoperiod, and light–dark temperature of 23 °C:18 °C, with relative humidity between 60 and 80%.

For virus treatments, we used a CABYV strain provided by H. Lecoq (INRA Avignon, France) collected from zucchini squash in Montfavet, France, in 2003. CABYV was maintained in *C. melo* cv. C-311 by serial passages using viruliferous *A. gossypii* previously reared on a CABYV-infected melon. The CMV M6 strain (CMV hereafter) was collected from a melon crop in Tarragona (Spain) in 1996 and kindly provided by E. Moriones (EELM-CSIC, Spain). CMV was maintained in *Cucurbita pepo* cv. ‘Negro Belleza’ by serial mechanical passes. To generate CMV-infected source plants, a CMV-infected leaf was homogenised using a solution with 0.03 M disodium phosphate and 0.2 diethyldithiocarbamate acid (1:10 g fresh tissue:mL of buffer solution). This solution was used to mechanically inoculate melon receptor plants using carborundum. Virus-infected source plants were kept in a growth chamber with a 16 h photoperiod, and light–dark temperature of 24 °C:20 °C, with relative humidity between 60 and 80%.

### Plant material

A preliminary growth-chamber experiment compared three melon cultivars for their response to single and double inoculation with CMV and CABYV; the aim was to identify a cultivar with high susceptibility to both viruses to maximize the number of infected plants. Commercial hybrids with short cycles included two Galia-type cultivars, ‘Alcazaba’ (Rijk Zwaan Seeds, The Netherlands), ‘Buleria’ (Takii Seed, The Netherlands), and the Cantaloupe-type ‘Monique’ (Semillas Fitó, Barcelona, Spain). Apterous *A. gossypii* adults, reared as explained before, were used to inoculate the viruses. Virus treatments included a mock-inoculated control; single infection with either CMV or CABYV in the first true leaf; and double infections with CMV and CABYV, in the first and second leaf using the inoculation method described above. The experimental design was completely randomized with five replicates. For CMV, virus infection was determined observing symptoms 3 weeks after inoculation. To confirm CABYV virus infection, an enzyme-linked immunosorbent assay (ELISA)^55^ was conducted. A triple-antibody sandwich (TAS-ELISA) was conducted using CABYV antibodies (DSMZ, Germany), 6 weeks after virus inoculation. Virus transmission efficiencies were calculated as the number of infected plants divided by the number of plants inoculated. ‘Monique’ showed the highest susceptibility to both CMV and CABYV in single as well as in double infection (Supplementary Table S5). Therefore, this cultivar was chosen to conduct the experiment in the glasshouse described below. As a traditional cantaloupe-type short-cycle melon cultivar, ‘Monique’ develops within 70–90 days from germination to fruit harvesting.

### Plant husbandry and growing conditions

We used the phenological BBCH scale to score plant development^56^. Seeds were germinated on a filter paper over wet vermiculite in Petri dishes in a growth chamber with a 16 h photoperiod and light:dark temperature of 24 °C:20 °C. At stage 07, i.e., when radicle emerged and hypocotyl with open cotyledons breaking through the seed coat emerged, seedlings were transplanted to 15-cm diameter pots (12–15 seedlings per pot). At stage 11, i.e., 1-true emerging leaf, seedlings were taken to the greenhouse and individually transplanted to 30-cm diameter pots containing an autoclaved 1:2 mixture of vermiculite (No. 3, Asfaltex S.A., Spain) and soil substrate (Jiffy Tref GO V4, Castillo Arnedo S.L., Spain). At stage 14–15, i.e. 4–5 true leaves, we set a tripod with 2 m-long wooden stakes per pot joined at the top with adhesive tape for staking plants to the pots. A 1-cm depth sand layer was added to the top of the pot to reduce soil evaporation.

The glasshouse was set to day:night temperature of 26:20 °C and natural sunlight reduced with automatic shading when radiation exceeded 400 W·m^2^ from transplant to beginning of summer and set shading from 11 to 6 am from the beginning of summer until the end of the experiment. Actual temperature and relative humidity were monitored continuously with Tinytag ULTRA 2 data logger.

Irrigation regimes and method are explained below. Plants were weekly fertilized using 1 g of 20–20–20 (N:P:K) nutrient solution (Nutrichem 60, Miller Chemical & Fertilizer Corp., Hanover, USA) per plant. Three fungicides, Nimrod^®^ (Aragonesas Agro, Spain), Pelt^®^ (Bayer Cropscience, Germany) and Systhane^®^ (Corteva Agriscience, USA), were applied in rotation throughout the experiment to prevent powdery mildew.

### Virus inoculation

We established virus treatments at BBCH stage 17, i.e. 7-true leaves, 22 days after transplant. Groups of 20 apterous *A. gossypii* adults were used for each virus inoculation. Therefore, 20 aphids were used to mock-inoculate the control plants and single-CMV or single-CABYV infected plants, while 40 aphids (20 for CMV; 20 for CABYV) were used for double CMV + CABYV-infected plants. To avoid aphid escapes among the inoculated plants, viruliferous aphids were placed in a clip-cage over the adaxial side of the receptor leaf. For mock-inoculation, non-viruliferous aphids were used. For CMV-inoculation, aphids were starved for 1 h and later placed over a CMV-infected zucchini leaf for an acquisition access period (AAP) of 5 min, and later transferred to the 4th leaf from the bottom of the plants for an inoculation access period (IAP) of 48 h. For CABYV-infection, non-viruliferous *A. gossypii* adults were placed on CABYV-infected source plants for an AAP of 72 h. The day of the inoculation, aphids were collected from the virus-source plants and transferred to the 4^th^ leaf of the receptor plants for an IAP of 48 h. For double CMV and CABYV infection, we used the same method for virus acquisition and inoculation; to account for potential variation in plant response to the site of inoculation, 5 plants were inoculated following the sequence CMV in 4th and CABYV in 5th leaf and the other 5 plants following the reverse sequence, CABYV in the 4th leaf and CMV in the 5th leaf. After IAP, clip-cages and aphids were carefully removed using a hair brush from the plants that were then sprayed with Acetamiprid^®^ (Bayer Cropscience, Germany) (1 g/L) to kill any remaining aphids. Acetamiprid^®^ (1 g/L) was again applied preventively 55 days after transplanting (DAT) to avoid any insect pests on the plants.

### Water regimes

Plants within the 80% field capacity treatment were transplanted to pots at full water saturation (100% field capacity), and those within the 30% treatment, transplanted to pots at 50% of field capacity. After the transplant, water was deprived and pots regularly weighted until reaching either 80% or 30% of the field capacity. This point was then set as the beginning of the water regimes establishment (15 days after transplant; 7 days before virus inoculation). We determined the water-holding capacity of the substrate in the pots (herein pot capacity) with the following procedure. Sterilized soil and vermiculite were mixed in a 1:2 ratio and dried on greenhouse benches. All pots were filled with dry substrate to a set weight of 3 kg (soil plus pot). A sample of 5 pots was carefully irrigated from above and placed on trays containing water. After this overnight treatment, pots were placed over saucers to drain, and drained water was removed every half an hour. Once pots stopped draining, we determined pot capacity gravimetrically. Apparent plant water use was determined gravimetrically in three pots per treatment randomly picked at the beginning of the experiment. Later, another pot was added to the first three for a better estimation of the volume of irrigation to be applied. The frequency of measurements was 3 times a week throughout the experiment. Based on the apparent water use, plants were watered to maintain two water regimes, well-watered, i.e., 80%, and water stressed, i.e., 30%, of pot capacity. The assumptions of the gravimetric method to estimate plant transpiration are that soil evaporation and plant mass are negligible. A sand layer reduces evaporation, and plant mass is negligible for most of the growing season. Fruit weight late in the season was estimated for each of the four pots selected for each treatment.

### Experimental research involving plants

Our experimental research involving plants complies with all the relevant institutional, local, national, and international guidelines and legislation. The *Cucumis melo* cv. ‘Monique’ seeds were ordered from Semillas Fitó (Barcelona, Spain), being this cultivar one of the cantaloupe-type melon grown in cultivated areas in Spain. The aphid vector (*Aphis gossypii*), and plant viruses (cucumber mosaic virus: CMV; and cucurbit aphid-borne yellow virus: CABYV) used for virus inoculation in this experiment have been widely sampled and detected in several host plants in Spain.

### Measurements

#### Virus infection symptoms and ELISA

Virus infection symptoms in infected plants were assessed weekly from one week after virus infection until the end of the experiment to assess the sensitivity of plants (sensu Cooper and Jones^57^,) to virus infection. Plant symptoms induced by CABYV and CMV infections were visually assessed using a scale from 0 to 3: 0, no symptoms; 1, minor symptoms; 2, moderate symptoms; 3, severe symptoms and/or generalized infection (Fig. 1b). Symptom scoring in double infected plants was reliable because CMV and CABYV have sharp differences in type and location of symptoms. CMV-symptoms are chlorosis and mosaic mainly expressed in young, uppermost leaves while CABYV symptoms were yellowing of the entire, oldest bottom leaves. CMV-symptoms appeared earlier than those of CABYV in double infected plants.

To confirm virus infection we conducted an enzyme-linked immunosorbent assay (ELISA)^55^. For CMV, double-antibody sandwich (DAS-ELISA) was conducted using CMV antibodies (Bioreba, Switzerland) 4 weeks after virus infection. For CABYV, triple-antibody sandwich (TAS-ELISA) was conducted using CABYV antibodies (DSMZ, Germany) 6 weeks after virus infection. Similarly, two additional ELISA were conducted one week before final harvest: a DAS-ELISA for detection of CMV in either mock-inoculated or CABYV-infected plants, and a TAS-ELISA for detection of CABYV in any of the mock-inoculated or CMV-infected plants.

#### Leaf water potential

To characterize plant water status, we measured leaf water potential following methodology of Turner^58^, using a Scholander-type digital pressure chamber (Solfranc SF-20 Press; Solfranc Technologies, S.L., Spain). Briefly, a fully expanded, south-oriented leaf from top 6–10th node in the main stem was selected. The leaf was covered with a zip-type plastic bag before severing with a blade. A different blade was used for each leaf/treatment to avoid virus contamination among plants. Immediately after severing, the petiole of the bag-covered leaf was fitted into the chamber assembled for measurement. Pressure was slowly applied to the chamber and the leaf water potential was recorded when a small sap drop came out of the petiole. Measurements were taken in three plants per treatment, three times during the growing season: 31, 52 and 78 DAT.

#### Leaf temperature, greenness, and area

We measured leaf temperature, a surrogate for stomatal conductance, with an infrared thermometer (Tilswall, Model IR03B, USA). Leaf greenness, a surrogate for chlorophyll concentration, was measured with a chlorophyll meter (Opti-sciences, model CCM-200, USA). We measured the maximum leaf width using a ruler. Leaf area was calculated by applying the formula Area = π r^2^, assuming the leaf ratio as half of the leaf width, and the result later multiplied with a correction factor. To calculate the correction factor, the area of 15 randomly picked melon var. ‘Monique’ leaves was calculated using the formula. Later, each leaf was pictured and the real leaf area was calculated using Image J software. The coefficient was the result of the mean of the real area/π r^2^ calculations (Supplementary Table S2). Leaf greenness, temperature and area were measured weekly since 1-week after transplanting in three plants per treatment. Measurements were taken on the 1st leaf in the first week; on the 1st, 2nd and 3rd leaves in the second week; on the 3th and 4th in the third week; on the 7th leaf from the bottom for the fourth to fifth weeks, and on both the 7th leaf from the bottom the and 4th from the top from the sixth week until plant and harvest.

#### Dynamics of flowering, fruiting, and fruit set

Male flowers per plant were counted weekly from 25 to 36 days (3 counts) after transplant; using male flowers for pollination precluded reliable counting afterwards. Female flowers were counted every 2–3 days from the onset of their appearance until harvest. All female flowers were hand-pollinated by gently removing the petals from three male flowers and swabbing the pollen-covered anthers onto the lobes of the stigma of the female flower. Weekly, we counted fruit set and aborted fruit with characteristic yellowing and drying. Fruit set was calculated as the ratio between the number of melons produced and the total amount of female flowers.

#### Vegetative and reproductive traits at harvest

The experiment was harvested 78 DAT to the greenhouse when plants reached the fruit stage. Plants were separated in fruit, leaf lamina, and stem + petiole (hereafter “stem”) to determine fresh weight. Leaf and stem dry weight were measured after 48 h at 70 °C. Fruits were individually harvested and weighted using a digital scale (EK6015) and both fruit equatorial and polar diameter measured with a tailor’s tape measure. Fruit sugar concentration was measured in the ripest fruit of each plant by measuring the refractometric index (^o^Brix) at the middle point of the fruit flesh. We sampled the equatorial part with a 1 cm-diameter perforating punch, removed the skin, macerated the flesh, and collected several drops for sugar measurement with a refractometer (HHTEC, RHB-32ATC Model, Germany). Melon fruits were considered marketable when ^o^Brix was equal or greater than 10 degrees, following the maturity and ripeness criteria established for cantaloupe-type melons^59^.

#### Calculations and statistical analysis

We used JMP (version 17.2.0) to fitted linear models accounting for the experimental sources of variation—virus treatment, water regime, and their interaction, and the row-column effects to capture spatial variation for traits measured at harvest, and additionally accounting for repeated measures for traits measured regularly (Supplementary Tables 1–5). For traits measured several times, fitted curves allowed for patterns that were not apparent with analysis of individual dates, and allowed for parameters with standard errors that are interpretable biologically^60^. We used SigmaPlot (version 15) (Systat, Chicago, IL, USA) to fit non-linear models and least squares linear regressions, and IRENE^61^ to fit reduced minimum axis (RMA) regressions accounting for error in both *y* and *x*^62^. Allocation between leaf and stem was calculated in two ways: as a ratio, and as allometric coefficients to account for size-dependence^63^. Because *p* is not discrete, we present (i) the actual *p* from statistical models^64,65^ (ii) standard errors for comparison of treatments, and (iii) the complete statistical models in Supplementary Tables.

### Supplementary Information

Supplementary Information.

## References

[1] Han P (2019). Global change-driven modulation of bottom–up forces and cascading effects on biocontrol services. Curr. Opin. Insect Sci..

[2] Kankaanpää T (2020). Parasitoids indicate major climate-induced shifts in Arctic communities. Glob. Change Biol..

[3] Wootton JT (2002). Indirect effects in complex ecosystems: Recent progress and future challenges. J. Sea Res..

[4] Intergovermental panel on climate change 2022: Climate change 2022: Impacts, adaptation and vulnerability. Contribution of working group II to the sixth assessment report of the intergovernmental panel on climate change (ed. H.O. Pörtner, D.C. *et al*.) Cambridge University Press. Cambridge University Press, 3056 pp. (2022)

[5] Jones J (2016). Assessing recent trends in high-latitude Southern Hemisphere surface climate. Nat. Clim Chang..

[6] Jones RA, Naidu RA (2019). Global dimensions of plant virus diseases: Current status and future perspectives. Ann. Rev. Virol..

[7] Deutsch CA (2018). Increase in crop losses to insect pests in a warming climate. Science.

[8] Trebicki P (2020). Climate change and plant virus epidemiology. Virus Res..

[9] Lecoq H, Desbiez C (2012). Viruses of cucurbit crops in the Mediterranean region: An ever-changing picture. Adv. Virus Res..

[10] Moreno AB, López-Moya JJ (2020). When viruses play team sports: Mixed infections in plants. Phytopathology.

[11] Syller J (2014). Biological and molecular events associated with simultaneous transmission of plant viruses by invertebrate and fungal vectors. Mol. Plant Pathol..

[12] Piccinni G, Rush CM (2000). Determination of optimum irrigation regime and water use efficiency of sugar beet grown in pathogen-infested soil. Plant Dis..

[13] Avilla C, Collar JL, Duque M, Fereres A (1997). Yield of bell pepper (*Capsicum annuum*) inoculated with CMV and/or PVY at different time intervals. Z. Pflanzenkrankh. Pflanzenschutz J. Plant Dis. Prot..

[14] Juarez M (2013). Relative incidence, spatial distribution and genetic diversity of cucurbit viruses in eastern Spain. Ann. Appl. Biol..

[15] Körner CH, Mayr R, Grace J, Ford ED, Jarvis PG (1981). Stomatal behaviour in alpine plant communities between 600 and 2600 metres above sea level. Plants and their atmospheric environment.

[16] Slafer GA, Rawson HM (1994). Sensitivity of wheat phasic development to major environmental factors: A re-examination of some assumptions made by physiologists and modellers. Funct. Plant Biol..

[17] Hily JM, Poulicard N, Mora MÁ, Pagán I, García-Arenal F (2016). Environment and host genotype determine the outcome of a plant–virus interaction: From antagonism to mutualism. New Phytol..

[18] Pio-Ribeiro G, Wyatt SD, Kuhn CW (1978). Cowpea stunt: A disease caused by a synergistic interaction of two viruses. Phytopathology.

[19] Price JA (2010). Effects of wheat streak mosaic virus on root development and water-use efficiency of hard red winter wheat. Plant Dis..

[20] Xu P, Chen F, Mannas JP, Feldman T, Sumner LW, Roossinck MJ (2008). Virus infection improves drought tolerance. New Phytol..

[21] El Aou-Ouad H (2017). Combined effect of virus infection and water stress on water flow and water economy in grapevines. Physiol. Plant..

[22] Sadras V, Guirao M, Moreno A, Fereres AF (2023). Inter-virus relationships in mixed infections and virus- drought relationships in plants: A quantitative review. Plant J..

[23] Rabadán MP, Gómez P (2023). Global phylodynamics of two relevant aphid-transmitted viruses in cucurbit crops: Cucurbit aphid-borne yellows virus and watermelon mosaic virus. Phytopathol. Res..

[24] Escriu F, Fraile A, García-Arenal F (2003). The evolution of virulence in a plant virus. Evolution..

[25] El Aou-ouad H, Montero R, Medrano H, Bota J (2016). Interactive effects of grapevine leafroll-associated virus 3 (GLRaV-3) and water stress on the physiology of *Vitis vinifera* L. cv. Malvasia de Banyalbufar and Giro-Ros. J. Plant Physiol..

[26] Silva RGG (2016). Drought increases cowpea (*Vigna unguiculata* [L.] Walp) susceptibility to cowpea severe mosaic virus (CPSMV) at early stage of infection. Plant Physiol. Biochem..

[27] Mishra R (2022). Interplay between abiotic (drought) and biotic (virus) stresses in tomato plants. Mol. Plant Pathol..

[28] Shteinberg M (2021). Tomato yellow leaf curl virus (TYLCV) promotes plant tolerance to drought. Cells.

[29] Botto CS (2023). Tomato yellow leaf curl Sardinia virus increases drought tolerance of tomato. Int. J. Mol. Sci..

[30] van Munster M, Yvon M, Vile D, Dader B, Fereres A, Blanc S (2017). Water deficit enhances the transmission of plant viruses by insect vectors. PLoS ONE.

[31] Nachappa P, Culkin CT, Saya PM, Han JL, Nalam VJ (2016). Water stress modulates soybean aphid performance, feeding behavior, and virus transmission in soybean. Front. Plant Sci..

[32] Yvon M, Vile D, Brault V, Blanc S, van Munster M (2017). Drought reduces transmission of Turnip yellows virus, an insect-vectored circulative virus. Virus Res..

[33] Jones RAC (2005). Patterns of spread of two non-persistently aphid-borne viruses in lupin stands under four different infection scenarios. Ann. Appl. Biol..

[34] Hogenhout SA, Ammar D, Whitfield AE, Redinbaugh MG (2008). Insect vector interactions with persistently transmitted viruses. Ann. Rev. Phytopathol..

[35] Passioura JB (2006). The perils of pot experiments. Funct. Plant Biol..

[36] Jarvis PG, McNaughton K (1986). Stomatal control oftranspiration: Scaling up from leaf to region. Adv. Ecol. Res..

[37] Fereres E, Orgaz F, Gonzalez-Dugo V, Testi L, Villalobos FJ (2014). Balancing crop yield and water productivity tradeoffs in herbaceous and woody crops. Funct. Plant Biol..

[38] Fila G, Zeinalipour N, Badeck FWM, Delshad M, Ghashghaie J (2019). Application of water-saving treatments reveals different adaptation strategies in three Iranian melon genotypes. Sci. Hortic..

[39] Alonso-Prados J, Fraile A, Garcia-Arenal F (1997). Impact of cucumber mosaic virus and watermelon mosaic virus 2 infection on melon production in Central Spain. J. Plant Pathol..

[40] Davis TS, Bosque-Pérez NA, Foote NE, Magney T, Eigenbrode SD (2015). Environmentally dependent host–pathogen and vector–pathogen interactions in the Barley yellow dwarf viruspathosystem. J. Appl. Ecol..

[41] González R (2021). Plant virus evolution under strong drought conditions results in a transition from parasitism to mutualism. Proc. Natl. Acad. Sci..

[42] Mendez-Lozano J, Torres-Pacheco I, Fauquet CM, Rivera-Bustamante RF (2003). Interactions between geminiviruses in a naturally occurring mixture: Pepper huasteco virus and Pepper golden mosaic virus. Phytopathology.

[43] Palukaitis P, Roossinck MJ, Dietzgen RG, Francki RIB, Maramorosch K, Murphy FA, Shatkin AJ (1992). Cucumber mosaic virus. Advances in Virus Research.

[44] Porras MF (2020). Enhanced heat tolerance of viral-infected aphids leads to niche expansion and reduced interspecific competition. Nat. Commun..

[45] Stephenson AG (1981). Flower and fruit abortion: Proximate causes and ultimate functions. Ann. Rev. Ecol. Syst..

[46] Sakai S (2007). A new hypothesis for the evolution of overproduction of ovules: An advantage of selective abortion for females not associated with variation in genetic quality of the resulting seeds. Evolution.

[47] Porcher and Lande (2005). Reproductive compensations in the evolution of plant mating systems. New Phytol..

[48] Burd M (2009). Ovule number per flower in a world of unpredictable pollination. Am. J. Bot..

[49] Korbecka G, Klinkhamer PGL, Vrieling K (2002). Selective embryo abortionhypothesis revisited—A molecular approach. Plant Biol..

[50] Avilla C, Collar JL, Duque M, Fereres A (1997). Yield of bell pepper (*Capsicum annuum*) inoculated with CMV and/or PVY at different time intervals. J. Plant Dis. Protect..

[51] Kumar P (2022). Phloem connectivity and transport are not involved in mature plant resistance (MPR) to Potato Virus Y in different potato cultivars, and MPR does not protect tubers from recombinant strains of the virus. J. Plant Physiol..

[52] Anderson EJ, Kline AS, Morelock TE, McNew RW (1996). Tolerance to blackeye cowpea mosaic potyvirus not correlated with decreased virus accumulation or protection from cowpea stunt disease. Plant Dis..

[53] Tatineni S, Graybosch RA, Hein GL, Wegulo SN, French R (2010). Wheat cultivar-specific disease synergism and alteration of virus accumulation during co-infection with wheat streak mosaic virus and Triticum mosaic virus. Phytopathology.

[54] Brien CJ (2013). Accounting for variation in designing greenhouse experiments with special reference to greenhouses containing plants on conveyor systems. Plant Methods.

[55] Clark MF, Adams AN (1977). Characteristics of the microplate method of enzyme-linked immunosorbent assay for the detection of plant viruses. J. Gen. Virol..

[56] Lancashire PD (1991). A uniform decimal code for growth stages of crops and weeds. Ann. Appl. Biol..

[57] Cooper JL, Jones AT (1983). Responses of plants to viruses: Proposals for the use of terms. Phytopathology.

[58] Turner NC (1981). Technique and experimental approaches for the measurement of plant water status. Plant Soil..

[59] UNECE STANDARD FFV-23 concerning the marketing and commercial quality control of melons. https://unece.org/sites/default/files/2023-07/23_Melons_e.pdf (2017)

[60] Potvin C, Lechowicz MJ, Tardif S (1990). The statistical analysis of ecological response curves obtained from experiments involving repeated measures. Ecology.

[61] Fila G, Bellocchi G, Acutis M, Donatelli M (2003). Irene: A software to evaluate model performance. Eur. J. Agron..

[62] Ludbrook J (2012). A primer for biomedical scientists on how to execute Model II linear regression analysis. Clin. Exp. Pharmacol. Physiol..

[63] Poorter H, Sack L (2012). Pitfalls and possibilities in the analysis of biomass allocation patterns in plants. Front. Plant Sci..

[64] Ioannidis JPA (2019). What have we (not) learnt from millions of scientific papers with *P* values?. Am. Stat..

[65] Di Leo G, Sardanelli F (2020). Statistical significance: *p* value, 0.05 threshold, and applications to radiomics-reasons for a conservative approach. Eur. Radiol. Exp..

