# Supplementary material for: Interplay between drought and plant viruses co-infecting melon plants
Source: Sci Rep. 2024 Jul 9;14:15833. doi: 10.1038/s41598-024-66344-y (PMC11233556; doi:10.1038/s41598-024-66344-y)

**Supplementary Table S1:** Statistics of the fitted models for symptom vs time curves for well-watered and water-stressed plants infected with CMV, CMV+CABYV and CABYV.

| **CMV – Single infection – Well-watered** | | | | |  | **CMV – Single infection – Water-stressed** | | | | |
| --- | --- | --- | --- | --- | --- | --- | --- | --- | --- | --- |
| **R** | **Rsqr** | **Adj**  **Rsqr** | **Standard Error**  **of Estimate** | |  | **R** | **Rsqr** | **Adj**  **Rsqr** | **Standard Error**  **of Estimate** | |
| 0.9943 | 0.9886 | 0.9829 | 0.0808 | |  | 0.9899 | 0.9799 | 0.9699 | 0.1424 | |
|  | **Coeffi-**  **cient** | **Std.**  **Error** | **t** | **P** |  |  | **Coeffi-**  **cient** | **Std.**  **Error** | **t** | **P** |
| **Smax** | 2.3475 | 0.0765 | 30.6677 | <0.0001 |  | **Smax** | 2.2647 | 0.0875 | 25.8912 | <0.0001 |
| **b** | 8.3088 | 1.061 | 7.8311 | 0.0014 |  | **b** | 4.4059 | 0.8162 | 5.3982 | 0.0057 |
| **x0** | 37.1933 | 0.9684 | 38.4076 | <0.0001 |  | **x0** | 39.6414 | 1.0341 | 38.3352 | <0.0001 |

| **CMV – Double infection – Well-watered** | | | | |  | **CMV – Double infection – Water-stressed** | | | | |
| --- | --- | --- | --- | --- | --- | --- | --- | --- | --- | --- |
| **R** | **Rsqr** | **Adj**  **Rsqr** | **Standard Error**  **of Estimate** | |  | **R** | **Rsqr** | **Adj**  **Rsqr** | **Standard Error**  **of Estimate** | |
| 0.989 | 0.9782 | 0.9672 | 0.1818 | |  | 0.9845 | 0.9693 | 0.9539 | 0.1926 | |
|  | **Coeffi-**  **cient** | **Std.**  **Error** | **t** | **P** |  |  | **Coeffi-**  **cient** | **Std.**  **Error** | **t** | **P** |
| **Smax** | 3.0984 | 0.1328 | 23.3315 | <0.0001 |  | **Smax** | 2.9411 | 0.1317 | 22.338 | <0.0001 |
| **b** | 6.0413 | 1.0817 | 5.585 | 0.005 |  | **b** | 5.9239 | 1.2661 | 4.679 | 0.0095 |
| **x0** | 39.1974 | 1.215 | 32.2612 | <0.0001 |  | **x0** | 36.9646 | 1.3003 | 28.4287 | <0.0001 |

| **CABYV – Single infection – Well-watered** | | | | |  | **CABYV – Single infection – Water-stressed** | | | | |
| --- | --- | --- | --- | --- | --- | --- | --- | --- | --- | --- |
| **R** | **Rsqr** | **Adj**  **Rsqr** | **Standard Error**  **of Estimate** | |  | **R** | **Rsqr** | **Adj**  **Rsqr** | **Standard Error**  **of Estimate** | |
| 0.9939 | 0.9878 | 0.9816 | 0.1438 | |  | 0.9837 | 0.9677 | 0.9515 | 0.1897 | |
|  | **Coeffi-**  **cient** | **Std.**  **Error** | **t** | **P** |  |  | **Coeffi-**  **cient** | **Std.**  **Error** | **t** | **P** |
| **Smax** | 3.06 | 0.2026 | 15.1028 | 0.0001 |  | **Smax** | 2.1613 | 0.2696 | 8.018 | 0.0013 |
| **b** | 7.7369 | 1.2381 | 6.2491 | 0.0033 |  | **b** | 6.3856 | 1.9656 | 3.2486 | 0.0314 |
| **x0** | 48.4315 | 1.6489 | 29.3716 | <0.0001 |  | **x0** | 51.9201 | 2.6721 | 19.4303 | <0.0001 |

| **CABYV – Double infection – Well-watered** | | | | |  | **CABYV – Double infection – Water-stressed** | | | | |
| --- | --- | --- | --- | --- | --- | --- | --- | --- | --- | --- |
| **R** | **Rsqr** | **Adj**  **Rsqr** | **Standard Error**  **of Estimate** | |  | **R** | **Rsqr** | **Adj**  **Rsqr** | **Standard Error**  **of Estimate** | |
| 0.9993 | 0.9985 | 0.9978 | 0.0586 | |  | 0.9963 | 0.9926 | 0.9888 | 0.1245 | |
|  | **Coeffi-**  **cient** | **Std.**  **Error** | **t** | **P** |  |  | **Coeffi-**  **cient** | **Std.**  **Error** | **t** | **P** |
| **Smax** | 2.8721 | 0.0418 | 68.7381 | <0.0001 |  | **Smax** | 3.006 | 0.1442 | 20.8404 | <0.0001 |
| **b** | 3.8857 | 0.2721 | 14.2804 | 0.0001 |  | **b** | 6.1744 | 0.853 | 7.2384 | 0.0019 |
| **x0** | 46.6365 | 0.2969 | 157.083 | <0.0001 |  | **x0** | 49.4551 | 1.0705 | 46.1968 | <0.0001 |

**Supplementary Table S2:** Statistics of the fitted models for parameters measured on melon leaves: leaf water potential, leaf temperature, leaf greenness and leaf area. Data regarding the leaf area calibration experiment are indicated in the last table.

| **Leaf Water Potential** | | | | | | | | | |
| --- | --- | --- | --- | --- | --- | --- | --- | --- | --- |
| **Source** | | |  | | **Nparm** | **DF** | **DFDen** | **F Ratio** | **Prob > F** |
| Days after transplant | | | | 2 | | 2 | 40.93 | 4.0375 | 0.0251 |
| Water | | | | 1 | | 1 | 41.16 | 16.9624 | 0.0002 |
| Virus | | | | 3 | | 3 | 41.22 | 7.7141 | 0.0003 |
| Days after transplant*water | | | | 2 | | 2 | 40.93 | 0.4937 | 0.614 |
| Days after transplant*virus | | | | 6 | | 6 | 40.93 | 1.7821 | 0.1269 |
| Days after transplant*water*virus | | | | 6 | | 6 | 40.93 | 0.5818 | 0.7427 |
| Water*virus | | | | 3 | | 3 | 41.33 | 1.396 | 0.2575 |
|  |  |  | | |  |  |  |  |  |
| \| **Random Effect** \| \| --- \| \|  \| | **Var**  **Ratio** | **Var Component** | | | **Std**  **Error** | **95%**  **Lower** | **95%**  **Upper** | **Wald**  **p-Value** | **Pct of Total** |
| row | 0.0039161 | 3.07E-05 | | | 0.000117 | -0.000199 | 0.00026 | 0.7929 | 0.39 |
| column | -0.000843 | -6.61E-06 | | | 1.46E-06 | -9.48E-06 | -3.75E-06 | <.0001 | 0 |
| Residual |  | 0.0078426 | | | 0.0017335 | 0.005308 | 0.0127579 |  | 99.61 |
| Total |  | 0.0078733 | | | 0.0017255 | 0.0053448 | 0.0127484 |  | 100 |
|  |  |  | | |  |  |  |  |  |
| **RSquare** | **RSquare Adj** | **Root Mean Square Error** | | | **Mean of Response** | **Observations**  **(or Sum Wgts)** | |  |  |
| 0.616323 | 0.406214 | 0.088558 | | | 0.260606 | 66 | |  |  |

| **Leaf temperature (bottom leaf)** | | | | | | | | | | |
| --- | --- | --- | --- | --- | --- | --- | --- | --- | --- | --- |
| **Source** | | |  | | **Nparm** | | **DF** | **DFDen** | **F Ratio** | **Prob > F** |
| Days after transplant | | | | 10 | | | 10 | 241 | 34.1292 | <.0001 |
| Water | | | | 1 | | | 1 | 241 | 12.473 | 0.0005 |
| Virus | | | | 3 | | | 3 | 241.1 | 14.5872 | <.0001 |
| Days after transplant*water | | | | 10 | | | 10 | 241 | 0.715 | 0.7101 |
| Days after transplant*virus | | | | 30 | | | 30 | 241 | 3.306 | <.0001 |
| Days after transplant*water*virus | | | | 30 | | | 30 | 241 | 2.0389 | 0.0018 |
| Water*virus | | | | 3 | | | 3 | 241 | 6.825 | 0.0002 |
|  |  |  | | | |  |  |  |  |  |
| \| **Random Effect** \| \| --- \| \|  \| | **Var**  **Ratio** | **Var Component** | | | | **Std**  **Error** | **95%**  **Lower** | **95%**  **Upper** | **Wald**  **p-Value** | **Pct of Total** |
| row | 0.0320028 | 0.0406752 | | | | 0.0584491 | -0.073883 | 0.1552334 | 0.4865 | 3.101 |
| column | -0.000337 | -0.000428 | | | | 3.90E-05 | -0.000504 | -0.000352 | <.0001 | 0 |
| Residual |  | 1.2709906 | | | | 0.1157841 | 1.0714145 | 1.5323526 |  | 96.899 |
| Total |  | 1.3116659 | | | | 0.1296474 | 1.0905036 | 1.6080519 |  | 100 |
|  |  |  | | | |  |  |  |  |  |
| **RSquare** | **RSquare Adj** | **Root Mean Square Error** | | | | **Mean of Response** | **Observations**  **(or Sum Wgts)** | |  |  |
| 0.737906 | 0.643682 | 1.127382 | | | | 20.97939 | 330 | |  |  |

| **Leaf temperature (top leaf)** | | | | | | | | | | |
| --- | --- | --- | --- | --- | --- | --- | --- | --- | --- | --- |
| **Source** | | |  | | **Nparm** | | **DF** | **DFDen** | **F Ratio** | **Prob > F** |
| Days after transplant | | | | 4 | | | 4 | 68.45 | 31.292 | <.0001 |
| Water | | | | 1 | | | 1 | 69 | 5.7504 | 0.0192 |
| Virus | | | | 3 | | | 3 | 68.48 | 11.1006 | <.0001 |
| Days after transplant*water*virus | | | | 12 | | | 12 | 68.45 | 0.5394 | 0.8813 |
| Days after transplant*water | | | | 4 | | | 4 | 68.45 | 2.901 | 0.028 |
| Water*virus | | | | 3 | | | 3 | 65.23 | 3.9574 | 0.0118 |
| Days after transplant*virus | | | | 12 | | | 12 | 68.45 | 2.5622 | 0.0073 |
|  |  |  | | | |  |  |  |  |  |
| \| **Random Effect** \| \| --- \| \|  \| | **Var**  **Ratio** | **Var Component** | | | | **Std**  **Error** | **95%**  **Lower** | **95%**  **Upper** | **Wald**  **p-Value** | **Pct of Total** |
| row | 0.0006748 | 0.0009589 | | | | 0.0044911 | -0.007844 | 0.0097614 | 0.8309 | 0.067 |
| column | -0.000729 | -0.001036 | | | | 0.0066372 | -0.014044 | 0.0119731 | 0.876 | 0 |
| Residual |  | 1.4209774 | | | | 0.2428913 | 1.0434683 | 2.04915 |  | 99.933 |
| Total |  | 1.4219363 | | | | 0.2427805 | 1.0445084 | 2.0496063 |  | 100 |
|  |  |  | | | |  |  |  |  |  |
| **RSquare** | **RSquare Adj** | **Root Mean Square Error** | | | | **Mean of Response** | **Observations**  **(or Sum Wgts)** | |  |  |
| 0.775542 | 0.650486 | 1.192048 | | | | 23.60818 | 110 | |  |  |

| **Leaf greeness (bottom leaf)** | | | | | | | | | | |
| --- | --- | --- | --- | --- | --- | --- | --- | --- | --- | --- |
| **Source** | | |  | | **Nparm** | | **DF** | **DFDen** | **F Ratio** | **Prob > F** |
| Days after transplant | | | | 10 | | | 10 | 270 | 137.347 | <.0001 |
| Water | | | | 1 | | | 1 | 270 | 323.8643 | <.0001 |
| Virus | | | | 3 | | | 3 | 270.3 | 6.4778 | 0.0003 |
| Days after transplant*water | | | | 10 | | | 10 | 270 | 17.2382 | <.0001 |
| Days after transplant*virus | | | | 30 | | | 30 | 270 | 2.0307 | 0.0017 |
| Water*virus | | | | 3 | | | 3 | 270.4 | 0.2411 | 0.8677 |
| \| **Random Effect** \| \| --- \| \|  \| | **Var**  **Ratio** | **Var Component** | | | | **Std**  **Error** | **95%**  **Lower** | **95%**  **Upper** | **Wald**  **p-Value** | **Pct of Total** |
| row | 0.0330566 | 0.3666005 | | | | 0.5325757 | -0.677229 | 1.4104296 | 0.4912 | 3.169 |
| column | 0.0101768 | 0.1128612 | | | | 0.1669619 | -0.214378 | 0.4401006 | 0.4991 | 0.976 |
| Residual |  | 11.09009 | | | | 0.9545301 | 9.4335685 | 13.227254 |  | 95.856 |
| Total |  | 11.569551 | | | | 1.1051745 | 9.6758698 | 14.08238 |  | 100 |
|  |  |  | | | |  |  |  |  |  |
| **RSquare** | **RSquare Adj** | **Root Mean SquareError** | | | | **Mean of Response** | **Observations**  **(or Sum Wgts)** | |  |  |
| 0.712684 | 0.852088 | 3.330179 | | | | 14.50273 | 330 | |  |  |

| **Leaf greeness (top)** | | | | | | | | | | |
| --- | --- | --- | --- | --- | --- | --- | --- | --- | --- | --- |
| **Source** | | |  | | **Nparm** | | **DF** | **DFDen** | **F Ratio** | **Prob > F** |
| Days after transplant | | | | 4 | | | 4 | 81 | 14.0013 | <.0001 |
| Water | | | | 1 | | | 1 | 81 | 104.4266 | <.0001 |
| Virus | | | | 3 | | | 3 | 81.5 | 1.6823 | 0.1773 |
| Days after transplant*water | | | | 4 | | | 4 | 81 | 2.0806 | 0.0909 |
| Days after transplant*virus | | | | 12 | | | 12 | 81 | 1.5004 | 0.1411 |
| Water*virus | | | | 3 | | | 3 | 81.21 | 1.1819 | 0.3219 |
| \| **Random Effect** \| \| --- \| \|  \| | **Var**  **Ratio** | **Var Component** | | | | **Std**  **Error** | **95%**  **Lower** | **95%**  **Upper** | **Wald**  **p-Value** | **Pct of Total** |
| row | -0.000931 | -0.013037 | | | | 0.0020486 | -0.017052 | -0.009022 | <.0001 | 0 |
| column | 0.0044287 | 0.0620198 | | | | 0.1201063 | -0.173384 | 0.2974237 | 0.6056 | 0.441 |
| Residual |  | 14.00398 | | | | 2.20055 | 10.524044 | 19.557935 |  | 99.559 |
| Total |  | 14.066 | | | | 2.2002159 | 10.583438 | 19.612137 |  | 100 |
|  |  |  | | | |  |  |  |  |  |
| **RSquare** | **RSquare Adj** | **Root Mean Square Error** | | | | **Mean of Response** | **Observations**  **(or Sum Wgts)** | |  |  |
| 0.712684 | 0.61808 | 3.742189 | | | | 16.18091 | 110 | |  |  |

| **Leaf area (top)** | | | | | | |
| --- | --- | --- | --- | --- | --- | --- |
| **Source** |  | | **Nparm** | **DF** | **F Ratio** | **Prob > F** |
| Days after transplant | | 4 | | 30.027 | 9.120 | <.001 |
| Water | | 1 | | 76.845 | 1.229 | .271 |
| Virus | | 3 | | 76.845 | 38.267 | <.001 |
| Days after transplant*water | | 4 | | 30.027 | .068 | .991 |
| Daysaftertransplant*virus | | 12 | | 30.027 | .514 | .889 |
| Water*virus | | 3 | | 76.845 | 3.519 | .019 |
| Days after transplant*water*virus | | 12 | | 30.027 | .073 | 1.000 |

| **Leaf area (bottom)** | | | | | | |
| --- | --- | --- | --- | --- | --- | --- |
| **Source** |  | | **Nparm** | **DF** | **F Ratio** | **Prob > F** |
| Days after transplant | | 9 | | 32.172 | 851.276 | <.001 |
| Water | | 1 | | 123.026 | 125.007 | <.001 |
| Virus | | 3 | | 122.929 | .976 | .407 |
| Days after transplant*water | | 9 | | 32.172 | 6.277 | <.001 |
| Days after transplant*virus | | 27 | | 30.777 | .403 | .991 |
| Water*virus | | 3 | | 122.929 | 12.045 | <.001 |
| Days after transplant*water*virus | | 27 | | 30.777 | 1.719 | .074 |

| **Experiment to determine the coefficient for leaf area calculation** | | | | |
| --- | --- | --- | --- | --- |
| **#Leaf** | **Width (cm)** | **Area=** π ***(width/2)^2^ (cm^2^)** | **Real Area (Image J) (cm^2^)** | **Coefficient** |
| **1** | 25.5 | 510.69 | 442.58 | 0.87 |
| **2** | 26.2 | 539.11 | 481.41 | 0.89 |
| **3** | 12 | 113.09 | 115.49 | 1.02 |
| **4** | 19.4 | 295.58 | 281.01 | 0.95 |
| **5** | 24 | 452.38 | 411.34 | 0.91 |
| **6** | 12.9 | 130.69 | 116.89 | 0.89 |
| **7** | 22 | 380.12 | 304.92 | 0.80 |
| **8** | 13.1 | 134.78 | 118.65 | 0.88 |
| **9** | 20.6 | 333.28 | 305.13 | 0.92 |
| **10** | 16.6 | 216.42 | 185.36 | 0.86 |
| **11** | 14.9 | 174.36 | 162.49 | 0.93 |
| **12** | 16 | 201.06 | 189.25 | 0.94 |
| **13** | 16.7 | 219.03 | 185.16 | 0.85 |
| **14** | 12.7 | 126.67 | 103.75 | 0.82 |
| **15** | 14 | 153.93 | 135.61 | 0.88 |
|  |  |  |  | **Mean=0.89** |

**Supplementary Table S3.** Statistics of flowering and fruit set dynamics.

| **Dynamics of number of flowers** | | | | | | | | | |
| --- | --- | --- | --- | --- | --- | --- | --- | --- | --- |
| **Source** | | |  | | **Nparm** | **DF** | **DFDen** | **F Ratio** | **Prob > F** |
| Water | | | | 1 | | 1 | 1040 | 357.3358 | <.0001 |
| Virus | | | | 3 | | 3 | 1040 | 44.9574 | <.0001 |
| Days after transplant | | | | 15 | | 15 | 1040 | 144.0438 | <.0001 |
| Water*virus*days after transplant | | | | 45 | | 45 | 1040 | 0.2779 | 1 |
| Water*virus | | | | 3 | | 3 | 1040 | 6.3576 | 0.0003 |
| Water*days after transplant | | | | 15 | | 15 | 1040 | 6.5225 | <.0001 |
| Virus*days after transplant | | | | 45 | | 45 | 1040 | 2.547 | <.0001 |
|  |  |  | | |  |  |  |  |  |
| \| **Random Effect** \| \| --- \| \|  \| | **Var**  **Ratio** | **Var Component** | | | **Std**  **Error** | **95%**  **Lower** | **95%**  **Upper** | **Wald**  **p-Value** | **Pct of Total** |
| row | 0.0086856 | 0.0541406 | | | 0.0780827 | -0.098899 | 0.2071799 | 0.4881 | 0.857 |
| column | 0.0044687 | 0.027855 | | | 0.0403239 | -0.051178 | 0.1068884 | 0.4897 | 0.441 |
| Residual |  | 6.2334122 | | | 0.2733537 | 5.7305192 | 6.8058865 |  | 98.702 |
| Total |  | 6.3154079 | | | 0.2870757 | 5.7884635 | 6.9181136 |  | 100 |
|  |  |  | | |  |  |  |  |  |
| **RSquare** | **RSquare Adj** | **Root Mean Square Error** | | | **Mean of Response** | **Observations**  **(or Sum Wgts)** | |  |  |
| 0.748155 | 0.717459 | 2.49668 | | | 5.374359 | 1170 | |  |  |

| **Dynamics of fruit setting** | | | | | | | | | | |
| --- | --- | --- | --- | --- | --- | --- | --- | --- | --- | --- |
| **Source** | | |  | | **Nparm** | | **DF** | **DFDen** | **F Ratio** | **Prob > F** |
| Days after transplant | | | | 4 | | | 4 | 324 | 53.6431 | <.0001 |
| Water | | | | 1 | | | 1 | 324.3 | 20.262 | <.0001 |
| Virus | | | | 3 | | | 3 | 324.8 | 6.3016 | 0.0004 |
| Water*virus*days after transplant | | | | 12 | | | 12 | 324 | 0.6893 | 0.7618 |
| Water*virus | | | | 3 | | | 3 | 324.9 | 2.3171 | 0.0755 |
| Water*days after transplant | | | | 4 | | | 4 | 324 | 6.3419 | <.0001 |
| Virus*days after transplant | | | | 12 | | | 12 | 324 | 4.2801 | <.0001 |
|  |  |  | | | |  |  |  |  |  |
| \| **Random Effect** \| \| --- \| \|  \| | **Var**  **Ratio** | **Var Component** | | | | **Std**  **Error** | **95%**  **Lower** | **95%**  **Upper** | **Wald**  **p-Value** | **Pct of Total** |
| row | 0.0031555 | 0.0115007 | | | | 0.0191097 | -0.025954 | 0.048955 | 0.5473 | 0.314 |
| column | 0.0006801 | 0.0024789 | | | | 0.0052425 | -0.007796 | 0.0127541 | 0.6363 | 0.068 |
| Residual |  | 3.644698 | | | | 0.2863739 | 3.1426279 | 4.2781741 |  | 99.618 |
| Total |  | 3.6586776 | | | | 0.2867829 | 3.1557612 | 4.2928666 |  | 100 |
|  |  |  | | | |  |  |  |  |  |
| **RSquare** | **RSquare Adj** | **Root Mean Square Error** | | | | **Mean of Response** | **Observations**  **(or Sum Wgts)** | |  |  |
| 0.53115 | 0.475061 | 1.909109 | | | | 2.887978 | 366 | |  |  |

**Supplementary Table S4.** Statistics of the fitted models for traits at harvest: shoot dry matter, percentage of water in leaf, percentage of water in stem, leaf:stem ratio, stem diameter, stem length, stem volume, stem density, content of sugar in the fruit (Brix), number of melons per plant, total fruit weight per plant, equatorial diameter, polar diameter, fruit weight, number of marketable fruits per treatment, weight of marketable fruit per plant, and harvest index.

| **Shoot Dry Matter** | | | | | | | | | | |
| --- | --- | --- | --- | --- | --- | --- | --- | --- | --- | --- |
| **Source** | | |  | | **Nparm** | | **DF** | **DFDen** | **F Ratio** | **Prob > F** |
| Water | | | | 1 | | | 1 | 63.94 | 53.8721 | <.0001 |
| Virus | | | | 3 | | | 3 | 64.14 | 17.8872 | <.0001 |
| Water*virus | | | | 3 | | | 3 | 64.06 | 0.8114 | 0.4922 |
|  |  |  | | | |  |  |  |  |  |
| \| **Random Effect** \| \| --- \| \|  \| | **Var**  **Ratio** | **Var Component** | | | | **Std**  **Error** | **95%**  **Lower** | **95%**  **Upper** | **Wald**  **p-Value** | **Pct of Total** |
| row | -0.000634 | -0.364632 | | | | 0.0645814 | -0.491209 | -0.238054 | <.0001 | 0 |
| column | 0.0002083 | 0.1197781 | | | | 2.4986027 | -4.777393 | 5.0169495 | 0.9618 | 0.021 |
| Residual |  | 574.91197 | | | | 101.82497 | 417.86507 | 841.1835 |  | 99.979 |
| Total |  | 575.03175 | | | | 101.53042 | 418.33239 | 840.27145 |  | 100 |
|  |  |  | | | |  |  |  |  |  |
| **RSquare** | **RSquare Adj** | **Root Mean Square Error** | | | | **Mean of Response** | **Observations**  **(or Sum Wgts)** | |  |  |
| 0.621536 | 0.580778 | 23.9773 | | | | 111.3014 | 73 | |  |  |

| **% Water in leaf** | | | | | | | | | | |
| --- | --- | --- | --- | --- | --- | --- | --- | --- | --- | --- |
| **Source** | | |  | | **Nparm** | | **DF** | **DFDen** | **F Ratio** | **Prob > F** |
| Water | | | | 1 | | | 1 | 63.19 | 59.1288 | <.0001 |
| Virus | | | | 3 | | | 3 | 63.46 | 10.0147 | <.0001 |
| Water*virus | | | | 3 | | | 3 | 63.54 | 2.4115 | 0.075 |
|  |  |  | | | |  |  |  |  |  |
| \| **Random Effect** \| \| --- \| \|  \| | **Var**  **Ratio** | **Var Component** | | | | **Std**  **Error** | **95%**  **Lower** | **95%**  **Upper** | **Wald**  **p-Value** | **Pct of Total** |
| row | 0.0005248 | 0.0016762 | | | | 0.0149259 | -0.027578 | 0.0309305 | 0.9106 | 0.052 |
| column | 0.0023624 | 0.0075461 | | | | 0.0183595 | -0.028438 | 0.04353 | 0.6811 | 0.236 |
| Residual |  | 3.1942104 | | | | 0.5696836 | 2.3169191 | 4.6872381 |  | 99.712 |
| Total |  | 3.2034326 | | | | 0.5674144 | 2.328311 | 4.6872517 |  | 100 |
|  |  |  | | | |  |  |  |  |  |
| **RSquare** | **RSquare Adj** | **Root Mean Square Error** | | | | **Mean of Response** | **Observations**  **(or Sum Wgts)** | |  |  |
| 0.604744 | 0.562178 | 1.787235 | | | | 82.87291 | 73 | |  |  |

| **% water in stem** | | | | | | | | | | |
| --- | --- | --- | --- | --- | --- | --- | --- | --- | --- | --- |
| **Source** | | |  | | **Nparm** | | **DF** | **DFDen** | **F Ratio** | **Prob > F** |
| Water | | | | 1 | | | 1 | 65 | 3.0284 | 0.0866 |
| Virus | | | | 3 | | | 3 | 65 | 12.9518 | <.0001 |
| Water*virus | | | | 3 | | | 3 | 65 | 0.9704 | 0.4122 |
|  |  |  | | | |  |  |  |  |  |
| \| **Random Effect** \| \| --- \| \|  \| | **Var**  **Ratio** | **Var Component** | | | | **Std**  **Error** | **95%**  **Lower** | **95%**  **Upper** | **Wald**  **p-Value** | **Pct of Total** |
| row | -0.000391 | -0.000866 | | | | 0.0001519 | -0.001164 | -0.000568 | <.0001 | 0 |
| column | -8.47E-05 | -0.000188 | | | | 3.29E-05 | -0.000252 | -0.000123 | <.0001 | 0 |
| Residual |  | 2.2143356 | | | | 0.3884202 | 1.6139989 | 3.2269543 |  | 100 |
| Total |  | 2.2143356 | | | | 0.3884202 | 1.6139989 | 3.2269543 |  | 100 |
|  |  |  | | | |  |  |  |  |  |
| **RSquare** | **RSquare Adj** | **Root Mean Square Error** | | | | **Mean of Response** | **Observations**  **(or Sum Wgts)** | |  |  |
| 0.406875 | 0.343 | 1.488064 | | | | 89.88894 | 73 | |  |  |

| **Leaf:stem ratio** | | | | | | | | | | |
| --- | --- | --- | --- | --- | --- | --- | --- | --- | --- | --- |
| **Source** | | |  | | **Nparm** | | **DF** | **DFDen** | **F Ratio** | **Prob > F** |
| Water | | | | 1 | | | 1 | 65 | 21.8998 | <.0001 |
| Virus | | | | 3 | | | 3 | 65 | 7.2018 | 0.0003 |
| Water*virus | | | | 3 | | | 3 | 65 | 5.8565 | 0.0013 |
|  |  |  | | | |  |  |  |  |  |
| \| **Random Effect** \| \| --- \| \|  \| | **Var**  **Ratio** | **Var Component** | | | | **Std**  **Error** | **95%**  **Lower** | **95%**  **Upper** | **Wald**  **p-Value** | **Pct of Total** |
| row | -0.000087 | -3.39E-06 | | | | 5.95E-07 | -4.56E-06 | -2.23E-06 | <.0001 | 0 |
| column | -0.000286 | -1.12E-05 | | | | 1.96E-06 | -0.000015 | -7.32E-06 | <.0001 | 0 |
| Residual |  | 0.0389951 | | | | 0.0068402 | 0.028423 | 0.0568276 |  | 100 |
| Total |  | 0.0389951 | | | | 0.0068402 | 0.028423 | 0.0568276 |  | 100 |
|  |  |  | | | |  |  |  |  |  |
| **RSquare** | **RSquare Adj** | **Root Mean Square Error** | | | | **Mean of Response** | **Observations**  **(or Sum Wgts)** | |  |  |
| 0.481363 | 0.425509 | 0.197472 | | | | 1.482876 | 73 | |  |  |

| **Stem Diameter** | | | | | | | | | | |
| --- | --- | --- | --- | --- | --- | --- | --- | --- | --- | --- |
| **Source** | | |  | | **Nparm** | | **DF** | **DFDen** | **F Ratio** | **Prob > F** |
| Water | | | | 1 | | | 1 | 64.02 | 57.9899 | <.0001 |
| Virus | | | | 3 | | | 3 | 64.04 | 0.9155 | 0.4385 |
| Water*virus | | | | 3 | | | 3 | 64.03 | 1.1107 | 0.3514 |
|  |  |  | | | |  |  |  |  |  |
| \| **Random Effect** \| \| --- \| \|  \| | **Var**  **Ratio** | **Var Component** | | | | **Std**  **Error** | **95%**  **Lower** | **95%**  **Upper** | **Wald**  **p-Value** | **Pct of Total** |
| row | -0.001282 | -6.64E-06 | | | | 1.17E-06 | -8.94E-06 | -4.34E-06 | <.0001 | 0 |
| column | 0.0088884 | 0.000046 | | | | 8.23E-05 | -0.000115 | 0.0002073 | 0.5762 | 0.881 |
| Residual |  | 0.0051751 | | | | 0.0009149 | 0.0037635 | 0.0075661 |  | 99.119 |
| Total |  | 0.0052211 | | | | 0.0009164 | 0.0038049 | 0.0076107 |  | 100 |
|  |  |  | | | |  |  |  |  |  |
| **RSquare** | **RSquare Adj** | **Root Mean Square Error** | | | | **Mean of Response** | **Observations**  **(or Sum Wgts)** | |  |  |
| 0.521 | 0.469415 | 0.071938 | | | | 0.861836 | 73 | |  |  |

| **Stem Length** | | | | | | | | | | |
| --- | --- | --- | --- | --- | --- | --- | --- | --- | --- | --- |
| **Source** | | |  | | **Nparm** | | **DF** | **DFDen** | **F Ratio** | **Prob > F** |
| Water | | | | 1 | | | 1 | 63.02 | 33.3591 | <.0001 |
| Virus | | | | 3 | | | 3 | 63.07 | 4.0865 | 0.0103 |
| Water*virus | | | | 3 | | | 3 | 63.13 | 0.591 | 0.6232 |
|  |  |  | | | |  |  |  |  |  |
| \| **Random Effect** \| \| --- \| \|  \| | **Var**  **Ratio** | **Var Component** | | | | **Std**  **Error** | **95%**  **Lower** | **95%**  **Upper** | **Wald**  **p-Value** | **Pct of Total** |
| row | 0.0306851 | 0.003686 | | | | 0.0056813 | -0.007449 | 0.0148212 | 0.5165 | 2.936 |
| column | 0.0145628 | 0.0017493 | | | | 0.0027615 | -0.003663 | 0.0071618 | 0.5264 | 1.393 |
| Residual |  | 0.1201225 | | | | 0.0214058 | 0.0871522 | 0.1762078 |  | 95.671 |
| Total |  | 0.1255577 | | | | 0.0222241 | 0.0912763 | 0.1836621 |  | 100 |
|  |  |  | | | |  |  |  |  |  |
| **RSquare** | **RSquare Adj** | **Root Mean Square Error** | | | | **Mean of Response** | **Observations**  **(or Sum Wgts)** | |  |  |
| 0.539789 | 0.490228 | 0.346587 | | | | 3.86863 | 73 | |  |  |

| **Stem volume** | | | | | | | | | | |
| --- | --- | --- | --- | --- | --- | --- | --- | --- | --- | --- |
| **Source** | | |  | | **Nparm** | | **DF** | **DFDen** | **F Ratio** | **Prob > F** |
| Water | | | | 1 | | | 1 | 63.5 | 65.4414 | <.0001 |
| Virus | | | | 3 | | | 3 | 63.82 | 2.699 | 0.0531 |
| Water*Virus | | | | 3 | | | 3 | 63.9 | 0.7964 | 0.5004 |
|  |  |  | | | |  |  |  |  |  |
| \| **Random Effect** \| \| --- \| \|  \| | **Var**  **Ratio** | **Var Component** | | | | **Std**  **Error** | **95%**  **Lower** | **95%**  **Upper** | **Wald**  **p-Value** | **Pct of Total** |
| row | 0.001681 | 4.4462278 | | | | 16.667234 | -28.22095 | 37.113407 | 0.7897 | 0.168 |
| column | -0.000628 | -1.661367 | | | | 4.0543675 | -9.607781 | 6.2850477 | 0.682 | 0 |
| Residual |  | 2644.9816 | | | | 470.62693 | 1919.8594 | 3877.4775 |  | 99.832 |
| Total |  | 2649.4279 | | | | 469.80809 | 1925.0215 | 3878.4367 |  | 100 |
|  |  |  | | | |  |  |  |  |  |
| **RSquare** | **RSquare Adj** | **Root Mean Square Error** | | | | **Mean of Response** | **Observations**  **(or Sum Wgts)** | |  |  |
| 0.53836 | 0.488645 | 51.42938 | | | | 231.1192 | 73 | |  |  |

| **Stem Density** | | | | | | | | | | |
| --- | --- | --- | --- | --- | --- | --- | --- | --- | --- | --- |
| **Source** | | |  | | **Nparm** | | **DF** | **DFDen** | **F Ratio** | **Prob > F** |
| Water | | | | 1 | | | 1 | 64.12 | 3.6381 | 0.061 |
| Virus | | | | 3 | | | 3 | 64.29 | 7.0785 | 0.0003 |
| Water*virus | | | | 3 | | | 3 | 64.18 | 0.8587 | 0.4672 |
|  |  |  | | | |  |  |  |  |  |
| \| **Random Effect** \| \| --- \| \|  \| | **Var**  **Ratio** | **Var Component** | | | | **Std**  **Error** | **95%**  **Lower** | **95%**  **Upper** | **Wald**  **p-Value** | **Pct of Total** |
| row | 0.0012724 | 4.31E-06 | | | | 3.29E-05 | -0.00006 | 0.0000687 | 0.8955 | 0.127 |
| column | -0.0006 | -2.04E-06 | | | | 3.61E-07 | -2.74E-06 | -1.33E-06 | <.0001 | 0 |
| Residual |  | 0.0033906 | | | | 0.0006008 | 0.0024641 | 0.0049619 |  | 99.873 |
| Total |  | 0.0033949 | | | | 0.0005978 | 0.0024717 | 0.0049553 |  | 100 |
|  |  |  | | | |  |  |  |  |  |
| **RSquare** | **RSquare Adj** | **Root Mean Square Error** | | | | **Mean of Response** | **Observations**  **(or Sum Wgts)** | |  |  |
| 0.304831 | 0.229967 | 0.058229 | | | | 0.202598 | 73 | |  |  |

| **Fruit sugar content (^o^Brix)** | | | | | | | | | |
| --- | --- | --- | --- | --- | --- | --- | --- | --- | --- |
| **Source** | | |  | **Nparm** | | **DF** | **DFDen** | **F Ratio** | **Prob > F** |
| Water | | | | 1 | | 1 | 62.25 | 1.4777 | 0.2287 |
| Virus | | | | 3 | | 3 | 62.42 | 0.7087 | 0.5505 |
| Water*Virus | | | | 3 | | 3 | 62.52 | 0.2348 | 0.8718 |
|  |  |  | | |  |  |  |  |  |
| \| **Random Effect** \| \| --- \| \|  \| | **Var**  **Ratio** | **Var Component** | | | **Std**  **Error** | **95%**  **Lower** | **95%**  **Upper** | **Wald**  **p-Value** | **Pct of Total** |
| row | 0.0285043 | 0.1363872 | | | 0.2136876 | -0.282433 | 0.5552072 | 0.5233 | 2.771 |
| column | -0.000897 | -0.004293 | | | 0.0075655 | -0.019121 | 0.0105354 | 0.5704 | 0 |
| Residual |  | 4.7848023 | | | 0.8608021 | 3.4617491 | 7.0471153 |  | 97.229 |
| Total |  | 4.9211895 | | | 0.8829406 | 3.5632907 | 7.2396499 |  | 100 |
|  |  |  | | |  |  |  |  |  |
| **RSquare** | **RSquare Adj** | **Root Mean Square Error** | | | **Mean of Response** | **Observations**  **(or Sum Wgts)** | |  |  |
| 0.182269 | 0.09283 | 2.187419 | | | 10.62639 | 72 | |  |  |

| **Number of melons per plant** | | | | | | | | | |
| --- | --- | --- | --- | --- | --- | --- | --- | --- | --- |
| **Source** | | |  | **Nparm** | | **DF** | **DFDen** | **F Ratio** | **Prob > F** |
| Water | | | | 1 | | 1 | 65 | 0.3532 | 0.5544 |
| Virus | | | | 3 | | 3 | 65 | 2.9121 | 0.041 |
| Water*Virus | | | | 3 | | 3 | 65 | 0.1436 | 0.9334 |
|  |  |  | | |  |  |  |  |  |
| \| **Random Effect** \| \| --- \| \|  \| | **Var**  **Ratio** | **Var Component** | | | **Std**  **Error** | **95%**  **Lower** | **95%**  **Upper** | **Wald**  **p-Value** | **Pct of Total** |
| row | -0.000249 | -9.03E-05 | | | 1.58E-05 | -0.000121 | -5.93E-05 | <.0001 | 0 |
| column | -0.000195 | -7.06E-05 | | | 1.24E-05 | -9.49E-05 | -4.63E-05 | <.0001 | 0 |
| Residual |  | 0.3627018 | | | 0.0636221 | 0.2643684 | 0.5285658 |  | 100 |
| Total |  | 0.3627018 | | | 0.0636221 | 0.2643684 | 0.5285658 |  | 100 |
|  |  |  | | |  |  |  |  |  |
| **RSquare** | **RSquare Adj** | **Root Mean Square Error** | | | **Mean of Response** | **Observations**  **(or Sum Wgts)** | |  |  |
| 0.127322 | 0.033341 | 0.602247 | | | 1.767123 | 73 | |  |  |

| **Total fruit weight per plant** | | | | | | | | | |
| --- | --- | --- | --- | --- | --- | --- | --- | --- | --- |
| **Source** | | |  | **Nparm** | | **DF** | **DFDen** | **F Ratio** | **Prob > F** |
| Water | | | | 1 | | 1 | 64.1 | 34.351 | <.0001 |
| Virus | | | | 3 | | 3 | 64.27 | 3.1122 | 0.0323 |
| Water*Actual virus | | | | 3 | | 3 | 63.96 | 1.2301 | 0.3061 |
|  |  |  | | |  |  |  |  |  |
| \| **Random Effect** \| \| --- \| \|  \| | **Var**  **Ratio** | **Var Component** | | | **Std**  **Error** | **95%**  **Lower** | **95%**  **Upper** | **Wald**  **p-Value** | **Pct of Total** |
| row | 0.0010075 | 39.527491 | | | 379.55063 | -704.3781 | 783.43305 | 0.9171 | 0.101 |
| column | -0.000566 | -22.20635 | | | 3.9376836 | -29.92406 | -14.48863 | <.0001 | 0 |
| Residual |  | 39231.685 | | | 6956.6584 | 28505.051 | 57430.106 |  | 99.899 |
| Total |  | 39271.212 | | | 6918.204 | 28588.534 | 57331.471 |  | 100 |
|  |  |  | | |  |  |  |  |  |
| **RSquare** | **RSquare Adj** | **Root Mean Square Error** | | | **Mean of Response** | **Observations**  **(or Sum Wgts)** | |  |  |
| 0.438034 | 0.377514 | 198.0699 | | | 1307.534 | 73 | |  |  |

| **Equatorial diameter of the melon (Fruit 1)** | | | | | | | | | |
| --- | --- | --- | --- | --- | --- | --- | --- | --- | --- |
| **Source** | | |  | **Nparm** | | **DF** | **DFDen** | **F Ratio** | **Prob > F** |
| Water | | | | 1 | | 1 | 62.75 | 9.218 | 0.0035 |
| Virus | | | | 3 | | 3 | 63.14 | 10.0174 | <.0001 |
| Water*virus | | | | 3 | | 3 | 63.29 | 0.9331 | 0.4301 |
|  |  |  | | |  |  |  |  |  |
| \| **Random Effect** \| \| --- \| \|  \| | **Var**  **Ratio** | **Var Component** | | | **Std**  **Error** | **95%**  **Lower** | **95%**  **Upper** | **Wald**  **p-Value** | **Pct of Total** |
| row | 0.0108487 | 0.0526143 | | | 0.0936778 | -0.130991 | 0.2362195 | 0.5744 | 1.073 |
| column | -0.000851 | -0.004126 | | | 0.0157733 | -0.035041 | 0.0267896 | 0.7937 | 0 |
| Residual |  | 4.849808 | | | 0.8708463 | 3.510753 | 7.1371158 |  | 98.927 |
| Total |  | 4.9024224 | | | 0.8755351 | 3.5545381 | 7.1980313 |  | 100 |
|  |  |  | | |  |  |  |  |  |
| **RSquare** | **RSquare Adj** | **Root Mean Square Error** | | | **Mean of Response** | **Observations**  **(or Sum Wgts)** | |  |  |
| 0.424884 | 0.362948 | 2.202228 | | | 37.88356 | 73 | |  |  |

| **Polar diameter of the melon (Fruit 1)** | | | | | | | | | |
| --- | --- | --- | --- | --- | --- | --- | --- | --- | --- |
| **Source** | | |  | **Nparm** | | **DF** | **DFDen** | **F Ratio** | **Prob > F** |
| Water | | | | 1 | | 1 | 64.13 | 2.6082 | 0.1112 |
| Virus | | | | 3 | | 3 | 64.22 | 6.3789 | 0.0007 |
| Water*virus | | | | 3 | | 3 | 64.44 | 0.3094 | 0.8185 |
|  |  |  | | |  |  |  |  |  |
| \| **Random Effect** \| \| --- \| \|  \| | **Var**  **Ratio** | **Var Component** | | | **Std**  **Error** | **95%**  **Lower** | **95%**  **Upper** | **Wald**  **p-Value** | **Pct of Total** |
| row | 0.0047227 | 0.0356554 | | | 0.0877627 | -0.136356 | 0.207667 | 0.6845 | 0.47 |
| column | -0.000754 | -0.005691 | | | 0.0010061 | -0.007663 | -0.003719 | <.0001 | 0 |
| Residual |  | 7.5497363 | | | 1.3347112 | 5.4903516 | 11.037961 |  | 99.53 |
| Total |  | 7.5853917 | | | 1.3328943 | 5.5260747 | 11.062185 |  | 100 |
|  |  |  | | |  |  |  |  |  |
| **RSquare** | **RSquare Adj** | **Root Mean Square Error** | | | **Mean of Response** | **Observations**  **(or Sum Wgts)** | |  |  |
| 0.274794 | 0.196695 | 2.747678 | | | 38.67808 | 73 | |  |  |

| **Weight of the melon (Fruit 1 weight)** | | | | | | | | | |
| --- | --- | --- | --- | --- | --- | --- | --- | --- | --- |
| **Source** | | |  | **Nparm** | | **DF** | **DFDen** | **F Ratio** | **Prob > F** |
| Water | | | | 1 | | 1 | 64.07 | 10.6414 | 0.0018 |
| Virus | | | | 3 | | 3 | 64.12 | 6.4607 | 0.0007 |
| Water*virus | | | | 3 | | 3 | 64.28 | 0.4363 | 0.7278 |
|  |  |  | | |  |  |  |  |  |
| \| **Random Effect** \| \| --- \| \|  \| | **Var**  **Ratio** | **Var Component** | | | **Std**  **Error** | **95%**  **Lower** | **95%**  **Upper** | **Wald**  **p-Value** | **Pct of Total** |
| row | 0.0108525 | 301.29858 | | | 576.87255 | -829.3508 | 1431.948 | 0.6015 | 1.074 |
| column | -0.000832 | -23.09441 | | | 4.082744 | -31.09644 | -15.09238 | <.0001 | 0 |
| Residual |  | 27763.168 | | | 4908.1093 | 20190.191 | 40590.257 |  | 98.926 |
| Total |  | 28064.467 | | | 4922.9657 | 20455.645 | 40898.839 |  | 100 |
|  |  |  | | |  |  |  |  |  |
| **RSquare** | **RSquare Adj** | **Root Mean Square Error** | | | **Mean of Response** | **Observations**  **(or Sum Wgts)** | |  |  |
| 0.350548 | 0.280607 | 166.6228 | | | 883 | 73 | |  |  |

| **Number of marketable fruit per plant** | | | | | | | | | |
| --- | --- | --- | --- | --- | --- | --- | --- | --- | --- |
| **Source** | | |  | **Nparm** | | **DF** | **DFDen** | **F Ratio** | **Prob > F** |
| Water | | | | 1 | | 1 | 63.13 | 0.3069 | 0.5816 |
| Virus | | | | 3 | | 3 | 63.47 | 1.2542 | 0.2977 |
| Water*virus | | | | 3 | | 3 | 63.55 | 0.5649 | 0.6401 |
|  |  |  | | |  |  |  |  |  |
| \| **Random Effect** \| \| --- \| \|  \| | **Var**  **Ratio** | **Var Component** | | | **Std**  **Error** | **95%**  **Lower** | **95%**  **Upper** | **Wald**  **p-Value** | **Pct of Total** |
| row | 0.0103985 | 0.0022083 | | | 0.0039566 | -0.005546 | 0.009963 | 0.5768 | 1.029 |
| column | -0.000761 | -0.000162 | | | 0.0002874 | -0.000725 | 0.0004018 | 0.5741 | 0 |
| Residual |  | 0.2123626 | | | 0.0378763 | 0.1540351 | 0.3116304 |  | 98.971 |
| Total |  | 0.2145709 | | | 0.0379469 | 0.1560254 | 0.3137547 |  | 100 |
|  |  |  | | |  |  |  |  |  |
| **RSquare** | **RSquare Adj** | **Root Mean Square Error** | | | **Mean of Response** | **Observations**  **(or Sum Wgts)** | |  |  |
| 0.123407 | 0.029004 | 0.460828 | | | 0.684932 | 73 | |  |  |

| **Weight of marketable fruit per plant** | | | | | | | | | |
| --- | --- | --- | --- | --- | --- | --- | --- | --- | --- |
| **Source** | | |  | **Nparm** | | **DF** | **DFDen** | **F Ratio** | **Prob > F** |
| Water | | | | 1 | | 1 | 41.06 | 3.5412 | 0.067 |
| Virus | | | | 3 | | 3 | 41.3 | 4.6876 | 0.0066 |
| Water*virus | | | | 3 | | 3 | 40.56 | 0.1252 | 0.9447 |
|  |  |  | | |  |  |  |  |  |
| \| **Random Effect** \| \| --- \| \|  \| | **Var**  **Ratio** | **Var Component** | | | **Std**  **Error** | **95%**  **Lower** | **95%**  **Upper** | **Wald**  **p-Value** | **Pct of Total** |
| row | 0.0043027 | 120.75287 | | | 404.85066 | -672.7398 | 914.24558 | 0.7655 | 0.428 |
| column | -0.000901 | -25.29343 | | | 5.5880242 | -36.24575 | -14.3411 | <.0001 | 0 |
| Residual |  | 28064.306 | | | 6200.1886 | 18997.803 | 45641.205 |  | 99.572 |
| Total |  | 28185.059 | | | 6176.3043 | 19134.132 | 45635.005 |  | 100 |
|  |  |  | | |  |  |  |  |  |
| **RSquare** | **RSquare Adj** | **Root Mean Square Error** | | | **Mean of Response** | **Observations**  **(or Sum Wgts)** | |  |  |
| 0.325656 | 0.213265 | 167.524 | | | 920.98 | 50 | |  |  |

| **Harvest Index** | | | | | | | | | |
| --- | --- | --- | --- | --- | --- | --- | --- | --- | --- |
| **Source** | | |  | **Nparm** | | **DF** | **DFDen** | **F Ratio** | **Prob > F** |
| Water | | | | 1 | | 1 | 64.1 | 3.5299 | 0.0648 |
| Virus | | | | 3 | | 3 | 64.19 | 10.8844 | <.0001 |
| Water*virus | | | | 3 | | 3 | 64.41 | 0.7277 | 0.5392 |
|  |  |  | | |  |  |  |  |  |
| \| **Random Effect** \| \| --- \| \|  \| | **Var**  **Ratio** | **Var Component** | | | **Std**  **Error** | **95%**  **Lower** | **95%**  **Upper** | **Wald**  **p-Value** | **Pct of Total** |
| row | 0.0059307 | 0.000013488 | | | 0.00003248 | -0.0000501 | 0.0000771 | 0.678 | 0.59 |
| column | -0.000789 | -1.80E-06 | | | 3.17E-07 | -2.42E-06 | -1.17E-06 | <.0001 | 0 |
| Residual |  | 0.0022742 | | | 0.0004021 | 0.0016538 | 0.0033251 |  | 99.41 |
| Total |  | 0.0022877 | | | 0.0004017 | 0.001667 | 0.0033352 |  | 100 |
|  |  |  | | |  |  |  |  |  |
| **RSquare** | **RSquare Adj** | **Root Mean Square Error** | | | **Mean of Response** | **Observations**  **(or Sum Wgts)** | |  |  |
| 0.36948 | 0.301578 | 0.048355 | | | 0.614518 | 73 | |  |  |

**Table S5**. Virus transmission efficiencies (infected plants/total plants tested) in three melon cultivars, Alcazaba, Buleria and Monique. Plants were inoculated at 1-true leaf stage with either single CMV or CABYV and double CMV and CABYV infection using the aphid *Aphis gossypii*.

| Virus treatment | | Virus transmission (infected plants /total test plants) | | |
| --- | --- | --- | --- | --- |
|  |  | cv. Alcazaba | cv. Bulería | cv. Monique |
| CMV | | 5/5 | 5/5 | 5/5 |
| CMV + CABYV | CMV | 5/5 | 5/5 | 5/5 |
|  | CABYV | 3/5 | 2/5 | 5/5 |
| CABYV | | 3/5 | 4/5 | 5/5 |

**Figure S1:** Pot distribution in the greenhouse. Blocks were SE-NW oriented. Plants within treatments were distributed spatially to assure presence of plants belonging to each treatment in all blocks, rows and columns. Distances are in meters. A total of 80 plants were tested: 40 well-watered (blue) and 40 water-stressed plants (red). Within each water regimen treatment, 10 plants were mock-inoculated controls (green), 10 were CMV-infected (white), 10 were double-inoculated (half white/black) and 10 were CABYV-inoculated (black).


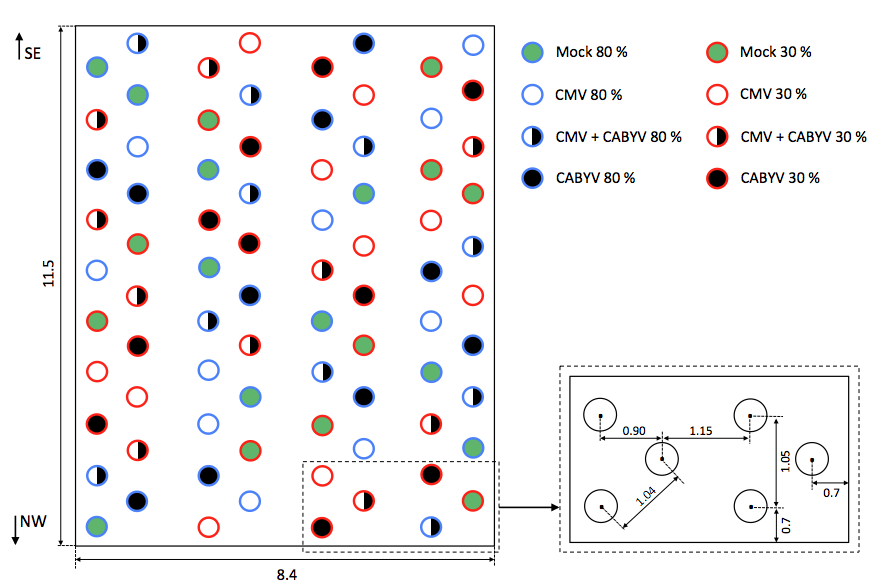

Supplement: Supplementary file 1 — Supplementary Information. [file 41598_2024_66344_MOESM1_ESM.docx]
